# Supplementary material for: Bis-Iridoid Glycosides and Triterpenoids from Kolkwitzia amabilis and Their Potential as Inhibitors of ACC1 and ACL
Source: Molecules. 2024 Dec 18;29(24):5980. doi: 10.3390/molecules29245980 (PMC11678491; doi:10.3390/molecules29245980)

Bis-iridoid glycosides and triterpenoids from *Kolkwitzia amabilis* and their potential as inhibitors of ACC1 and ACL

Jiang Wan [1], Ze-Yu Zhao [1,2], Can Wang [1], Chun-Xiao Jiang [1,2], Ying-Peng Tong [1], Yi Zang [3], Yeun-Mun Choo [4], Jia Li [3], Jin-Feng Hu [1,2,*]

[1] *School of Pharmaceutical Sciences, Zhejiang Provincial Key Laboratory of Plant Evolutionary Ecology and Conservation, Taizhou University, Taizhou 318000, PR China*

[2] *Department of Natural Medicine, School of Pharmacy, Fudan University, Shanghai 201203, PR China*

[3] *State Key Laboratory of Drug Research, Shanghai Institute of Materia Medica, Chinese Academy of Science, Shanghai 201203, PR China*

[4] *Chemistry Department, Faculty of Science, University of Malaya, 50603 Kuala Lumpur, Malaysia*

---

[*] Corresponding author at: Taizhou University, Taizhou 318000, PR China.

E-mail addresses: jfhu@tzc.edu.cn & jfhu@fudan.edu.cn (J.-F. Hu)

Contents

Figure S1. Phenylpropanoids from the twigs/leaves and flower buds of *K. amabilis*.

Figure S2. HPLC-ELSD profiles of monosaccharide hydrolysis of compound **7**.

Figures S3–S4. ^{1}H-^{1}H COSY, HMBC, and NOESY correlations of compounds **2** and **4**–**6**.

Figure S5. Molecular docking model of compound **17** bound to ACC1 and ACL.

Figure S6. Molecular docking model of compound **29** bound to ACL.

Figures S7–S13: 1D/2D NMR and HRESIMS spectra for compound **1**.

Figures S14–S18: 1D/2D NMR and HRESIMS spectra for compound **2**.

Figures S19–S25: 1D/2D NMR and HRESIMS spectra for compound **3**.

Figure S26: ECD spectrum of compound **3**.

Figures S27–S33: 1D/2D NMR and HRESIMS spectra for compound **4**.

Figure S34: ECD spectrum of compound **4**.

Figures S35–S40: 1D/2D NMR and HRESIMS spectra for compound **5**.

Figure S41: ECD spectrum of compound **5**.

Figures S42–S47: 1D/2D NMR and HRESIMS spectra for compound **6**.

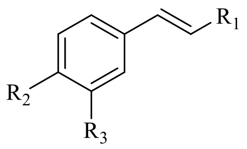

32: R_1 = CHO, R_2 = OMe, R_3 = OH
33: R_1 = COOH, R_2 = OMe, R_3 = OMe
34: R_1 = COOH, R_2 = OMe, R_3 = H
35: R_1 = COOH, R_2 = OH, R_3 = OMe
36: R_1 = CH_2OH, R_2 = *β*-D-Glu, R_3 = OMe

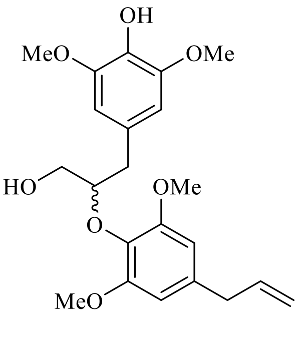

37

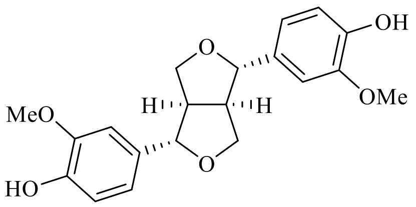

38

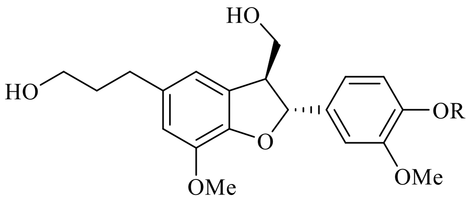

39: R = *β*-D-Glu

Figure S1. Phenylpropanoids (**32**–**39**) from the twigs/leaves and flower buds of *K. amabilis*.

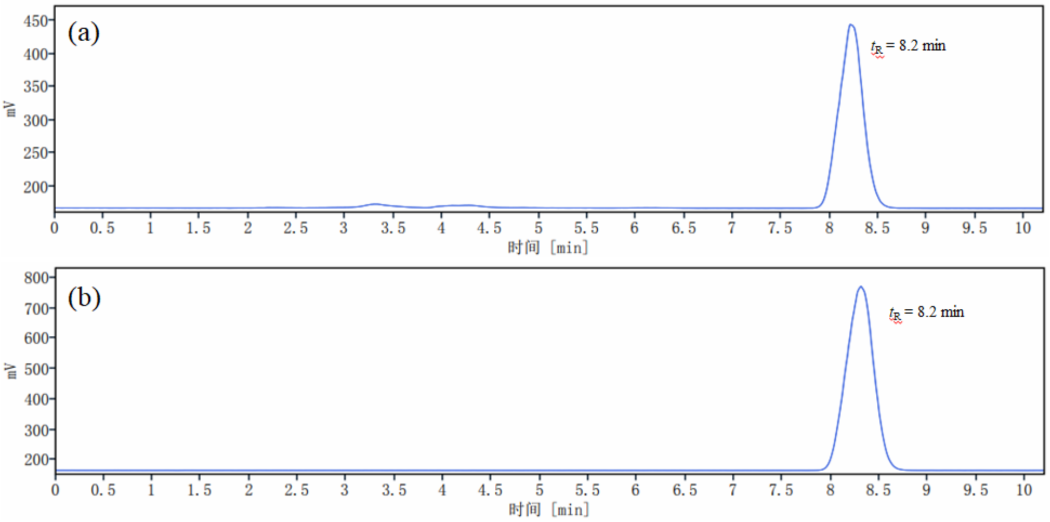

Figure S2. HPLC-ELSD profiles of monosaccharide hydrolysis of compound **7** (a: **7**; b: D-glucose).

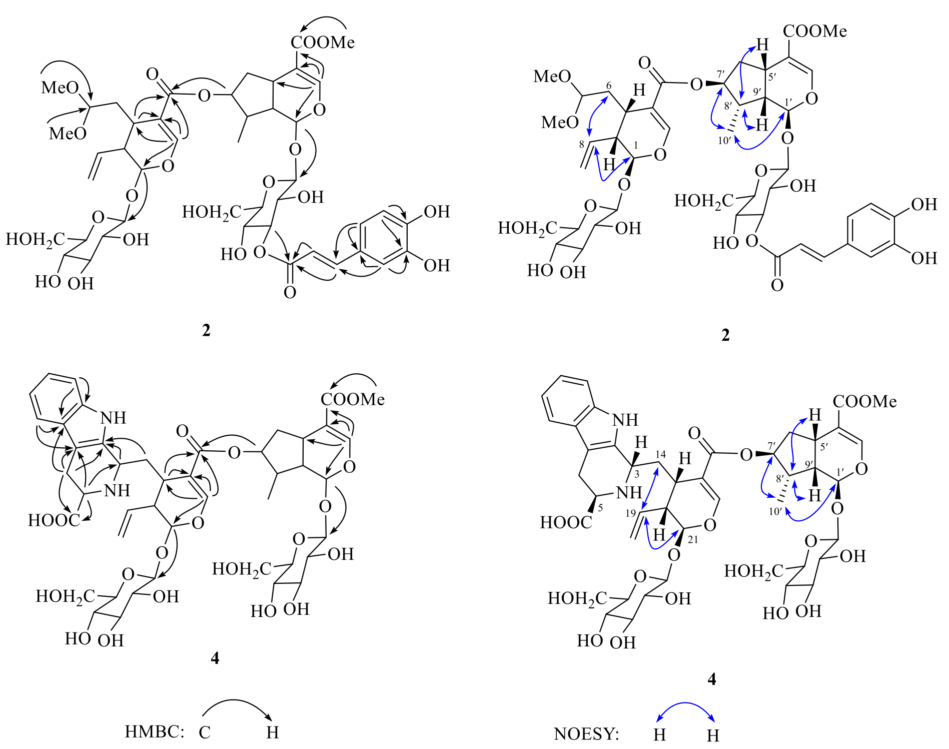

Figure S3. Observed key HMBC and NOESY correlations of **2** and **4**.

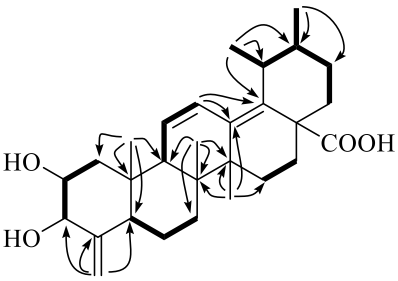

5

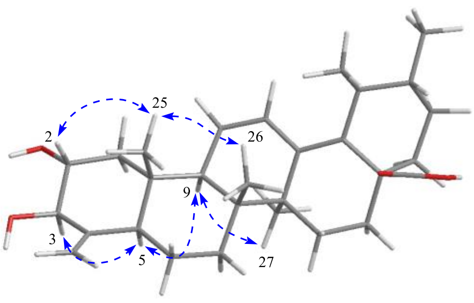

5

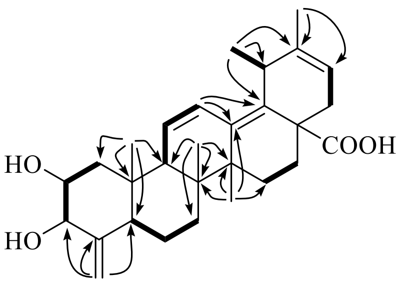

6

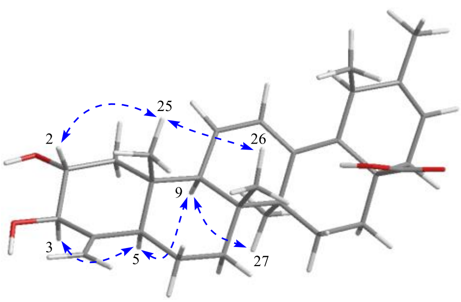

6

^{1}H-^{1}H COSY: ▬   HMBC: C ⌒ H   NOESY: H ⌒ H

Figure S4. ^{1}H-^{1}H COSY and observed key HMBC and NOESY correlations of **5** and **6**.

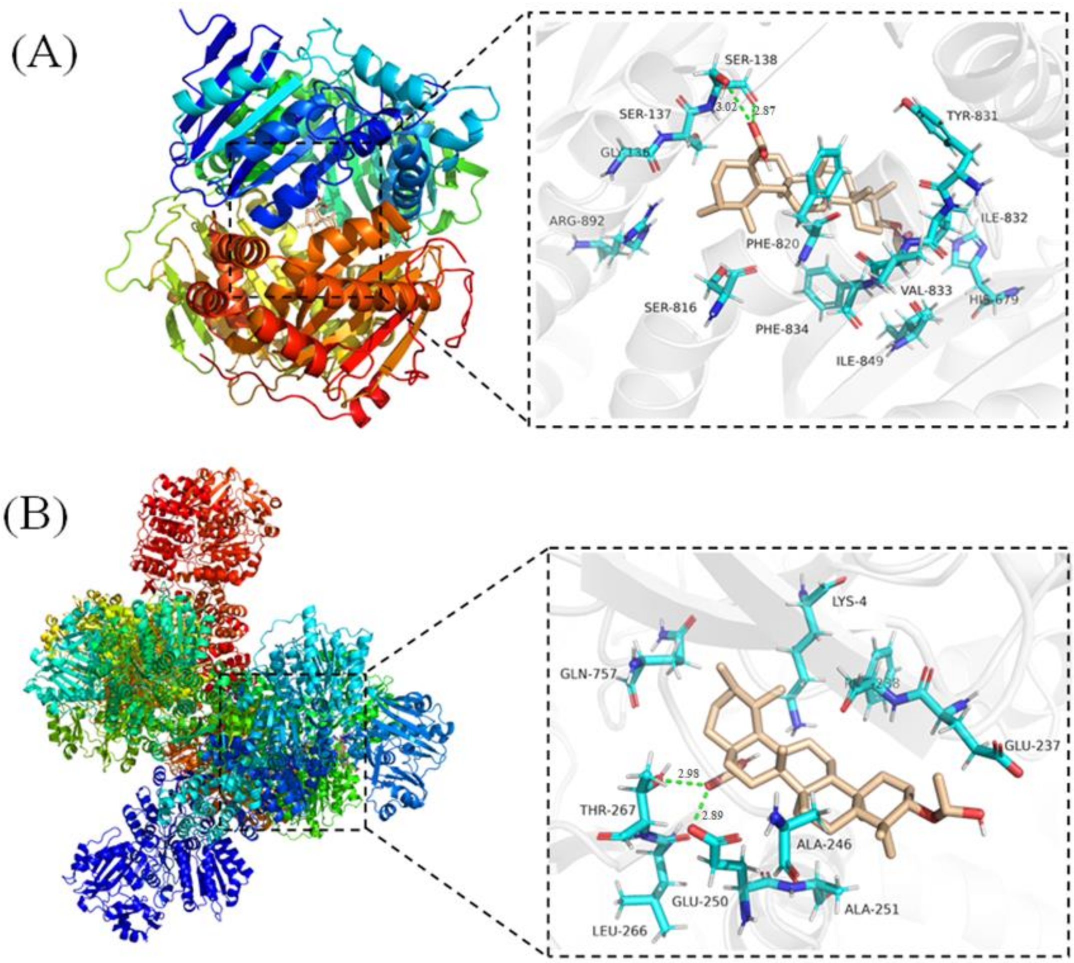

Figure S5. Molecular docking model of compound **17** bound to ACC1 (A, PDB ID: 3TVU) and ACL (B, PDB ID: 6HXH). These various interactions are displayed in dotted-lines (hydrogen bond: green).

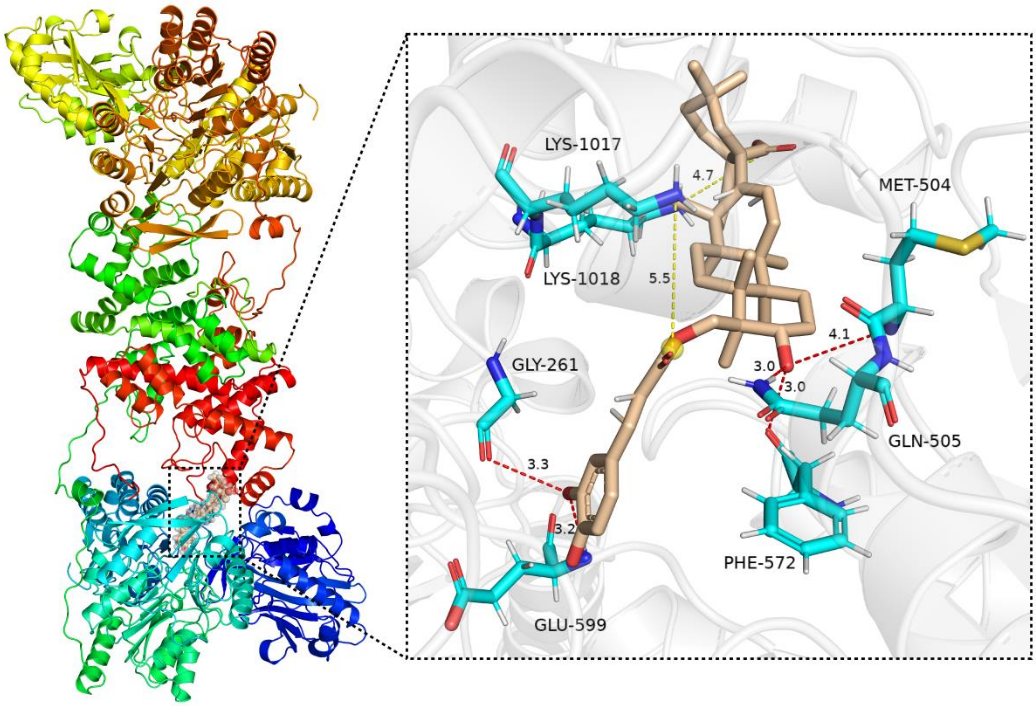

Figure S6. Molecular docking model of compound **29** bound to ACL (PDB ID: 6HXH). These various interactions are displayed in dotted-lines (hydrogen bond: red; salt bridge: yellow).

Figure S7. ^{1}H NMR spectrum of compound **1** in CD_3OD (400 MHz).

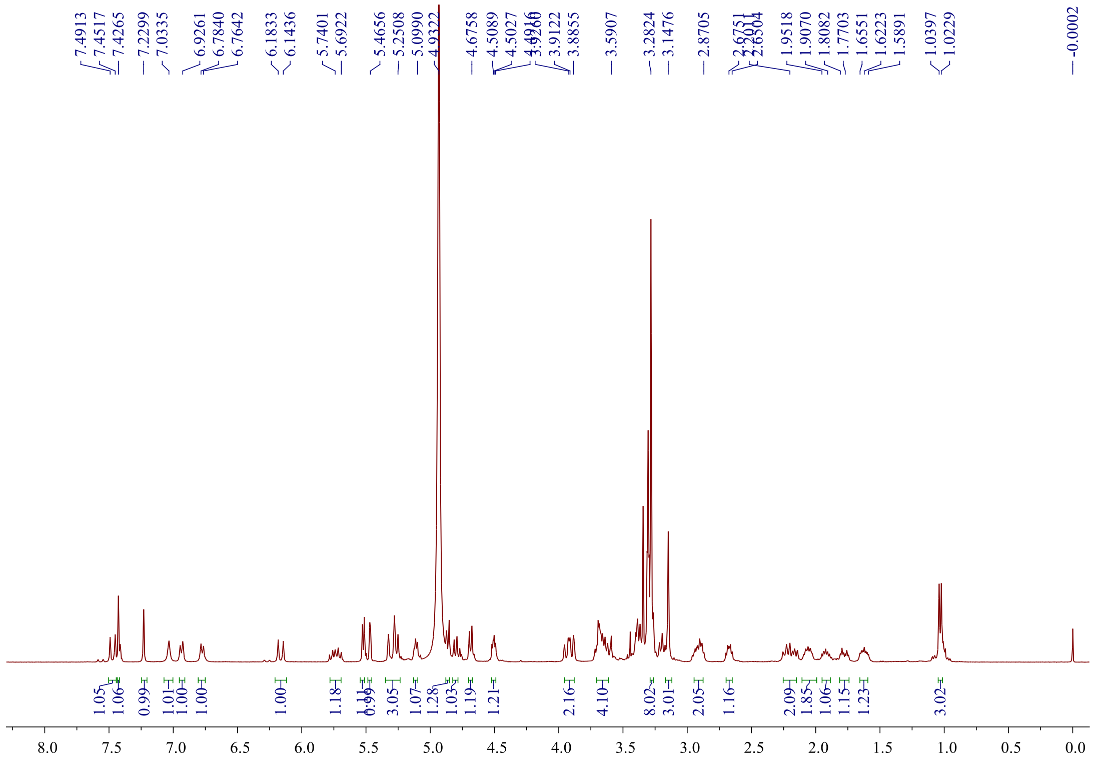

Figure S8. ^{13}C NMR spectra of compound **1** in CD_3OD (100 MHz).

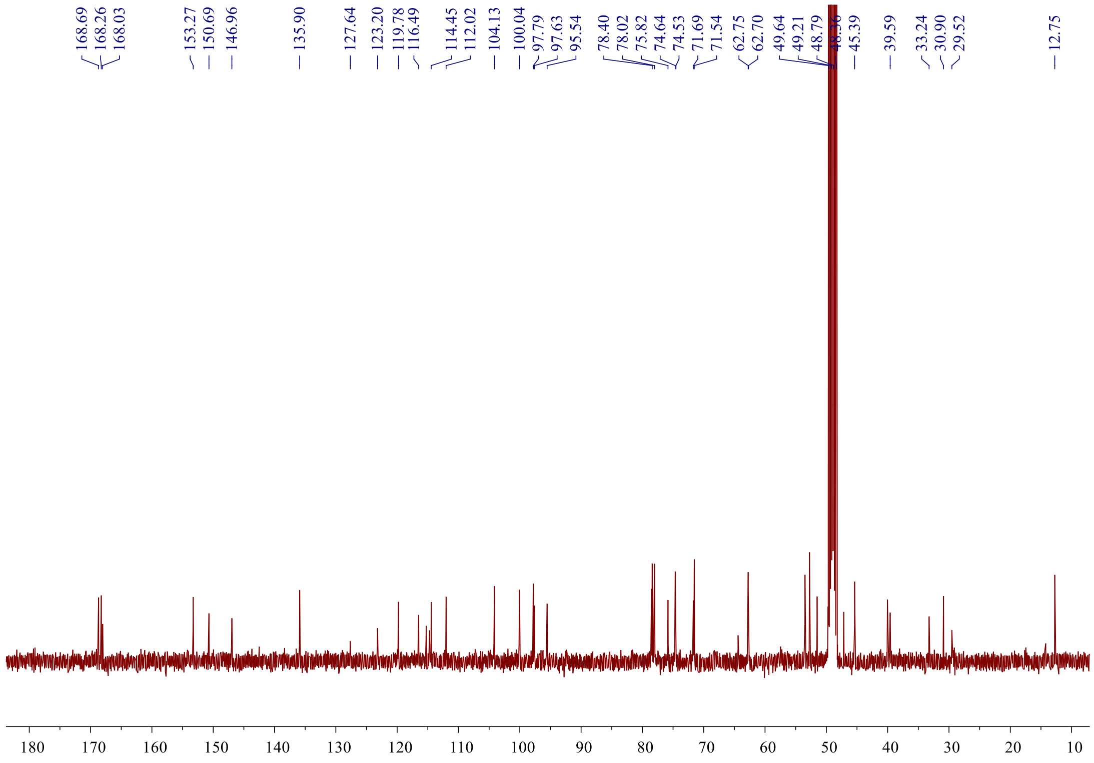

Figure S9. HSQC spectrum of compound **1** in CD_3OD (400 MHz).

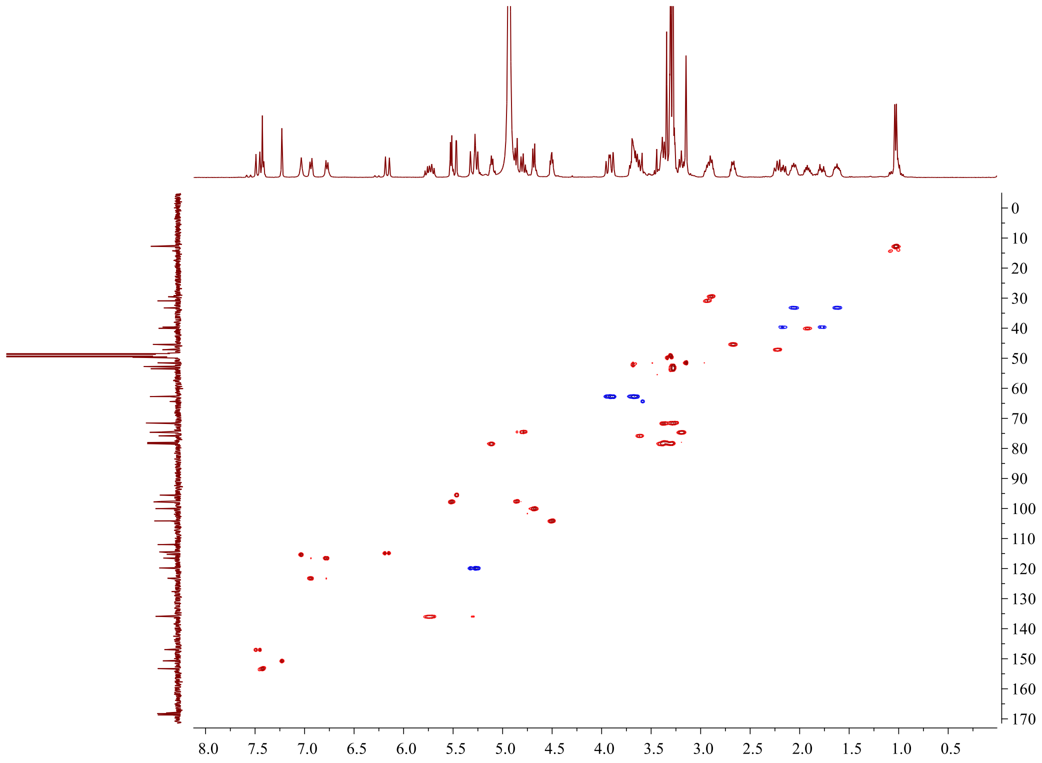

Figure S10. ^1H-^1H COSY spectrum of compound **1** in CD_3OD (400 MHz).

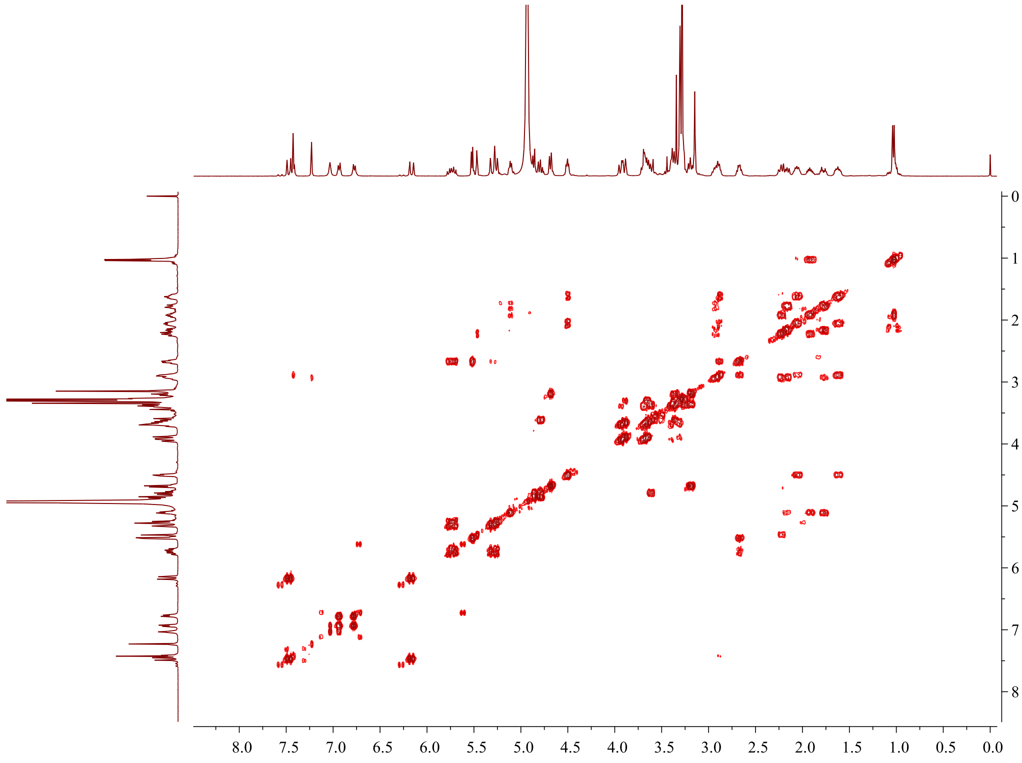

Figure S11. HMBC spectrum of compound **1** in CD_3OD (400 MHz).

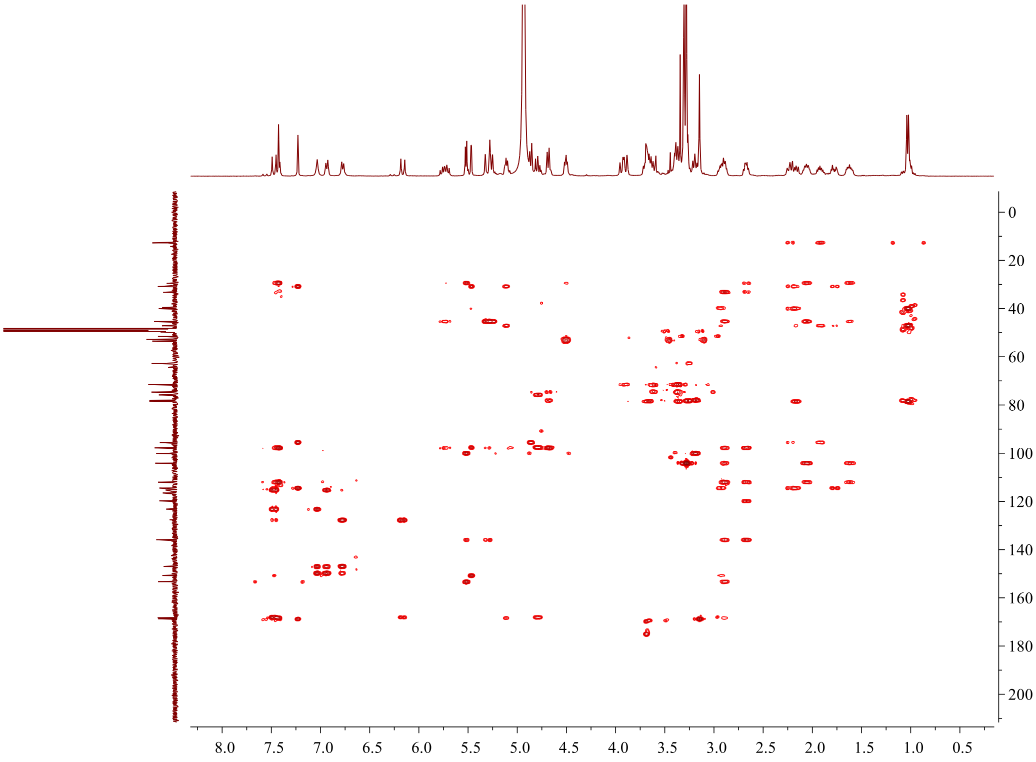

Figure S12. NOESY spectrum of compound **1** in CD_3OD (400 MHz).

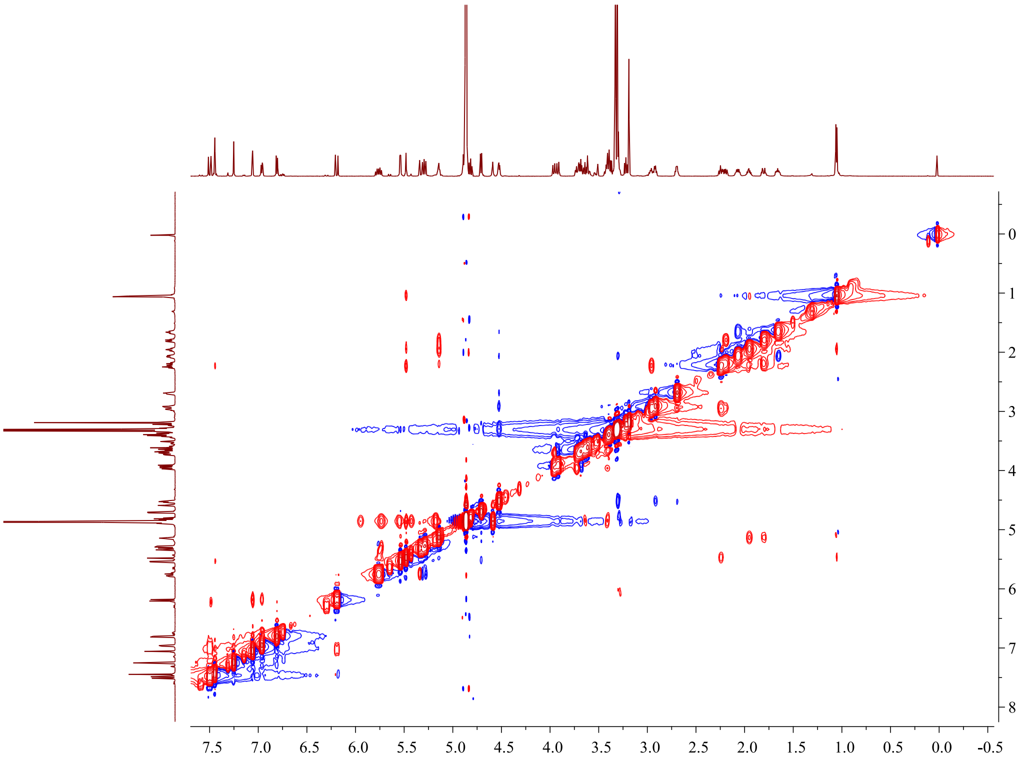

Figure S13. HRESIMS report of compound **1**.

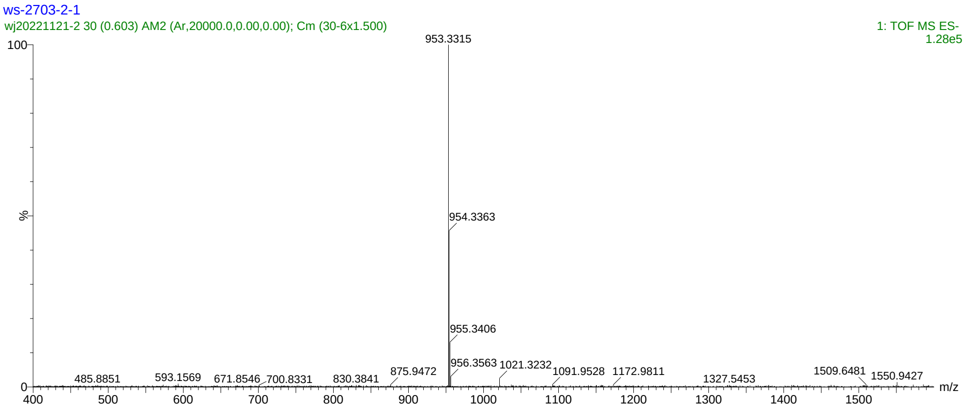

Figure S14. ^{1}H NMR spectrum of compound **2** in CD_3OD (400 MHz).

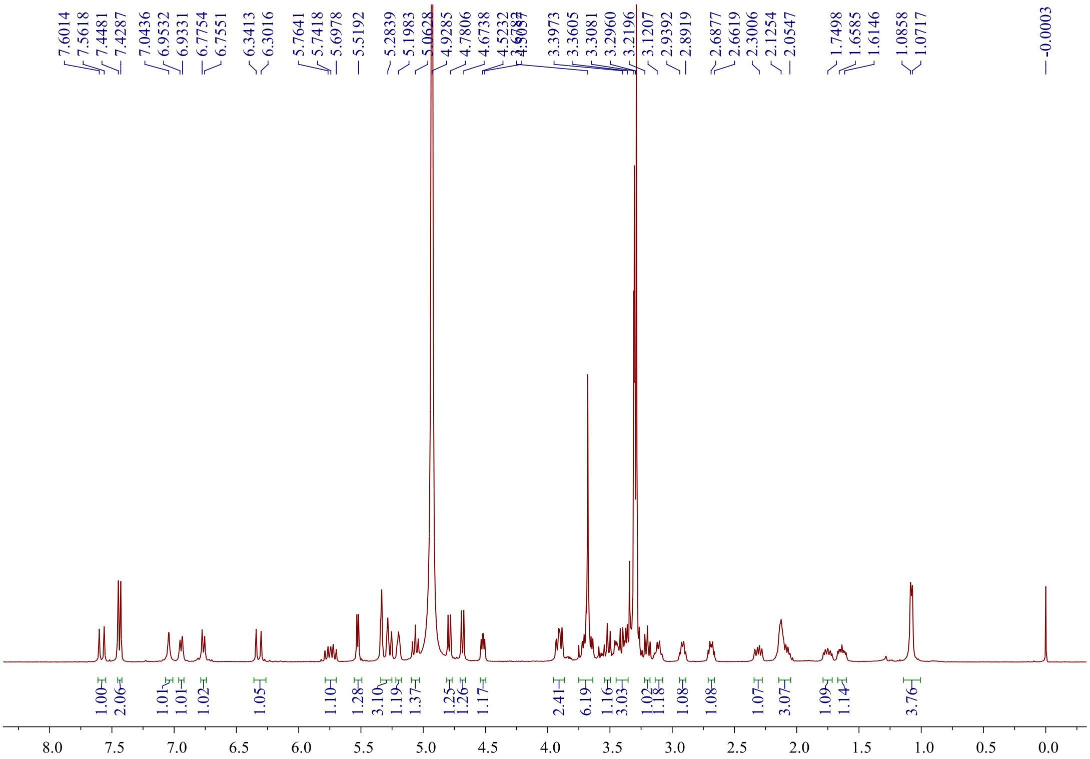

Figure S15. ^{13}C NMR spectrum of compound **2** in CD_3OD (100 MHz).

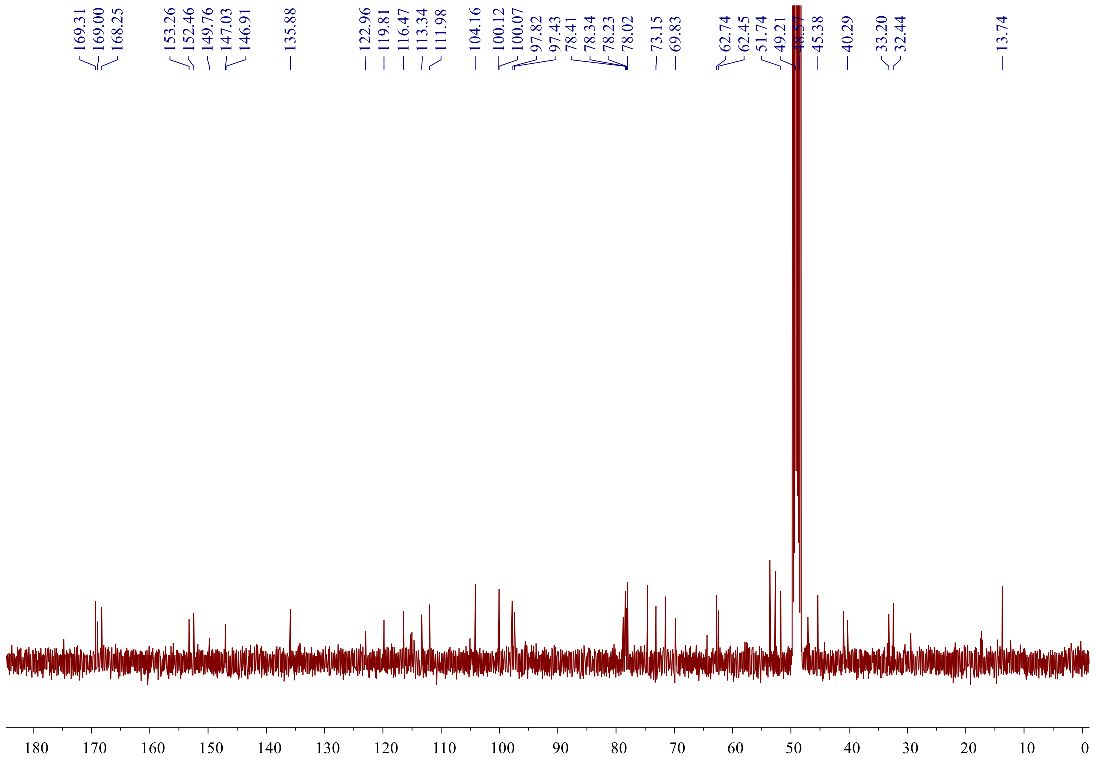

Figure S16. HMBC spectrum of compound **2** in CD_3OD (400 MHz).

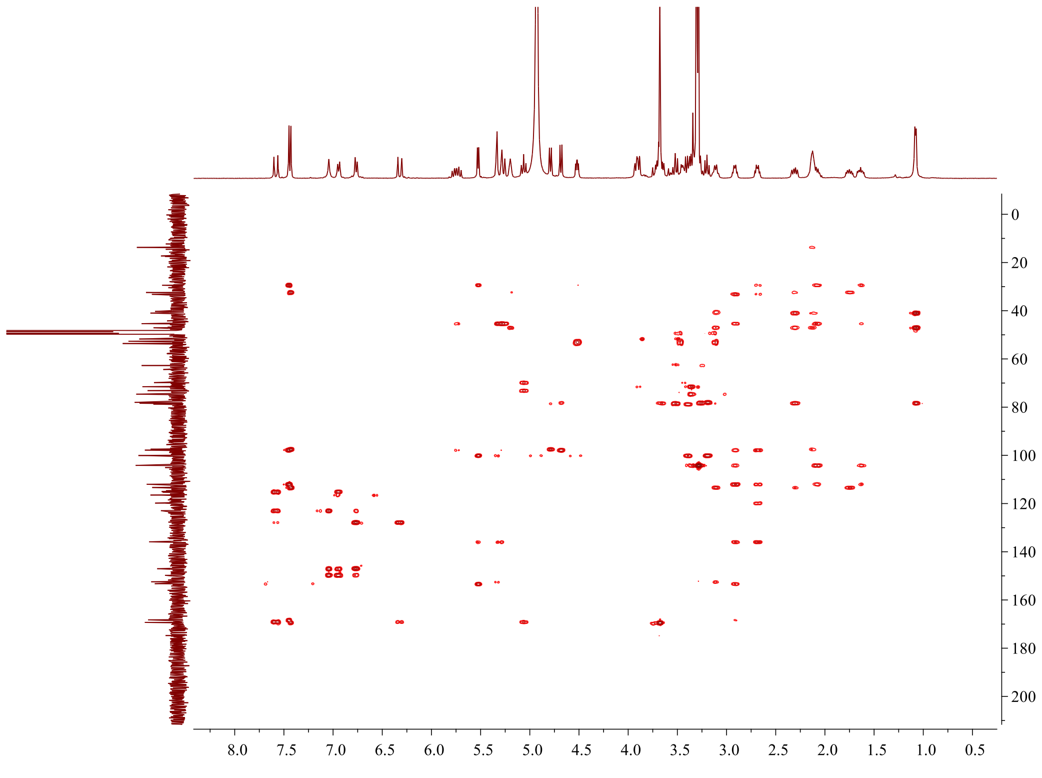

Figure S17. NOESY spectrum of compound **2** in CD_3OD (400 MHz).

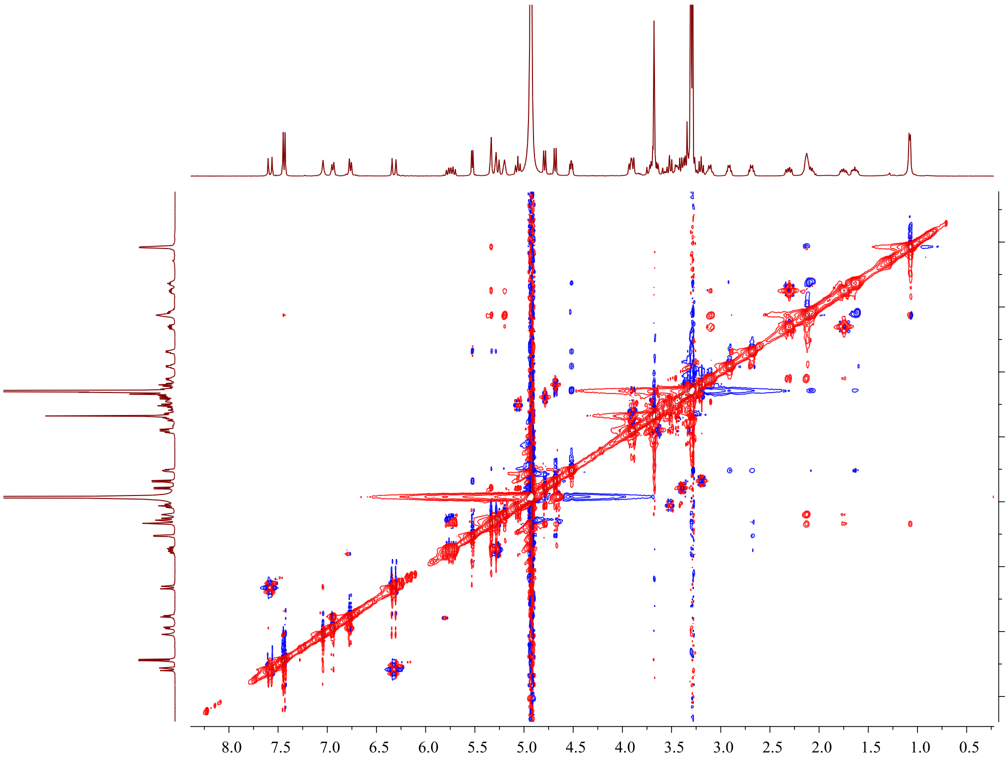

Figure S18. HRESIMS report of compound **2**.

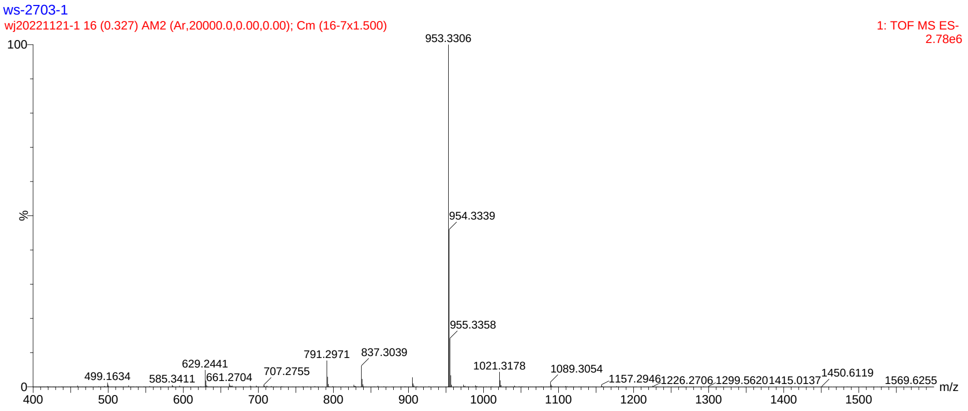

Figure S19. ^{1}H NMR spectrum of compound **3** in CD_3OD (400 MHz).

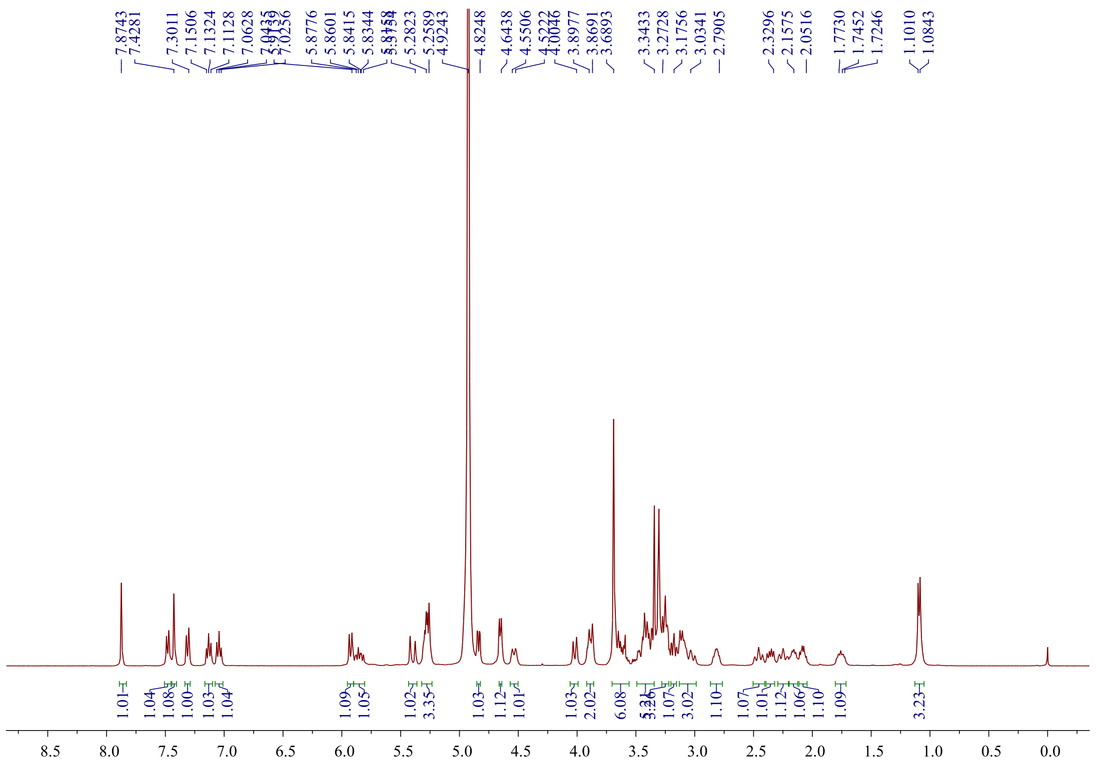

Figure S20. ^{13}C NMR spectrum of compound **3** in CD_3OD (100 MHz).

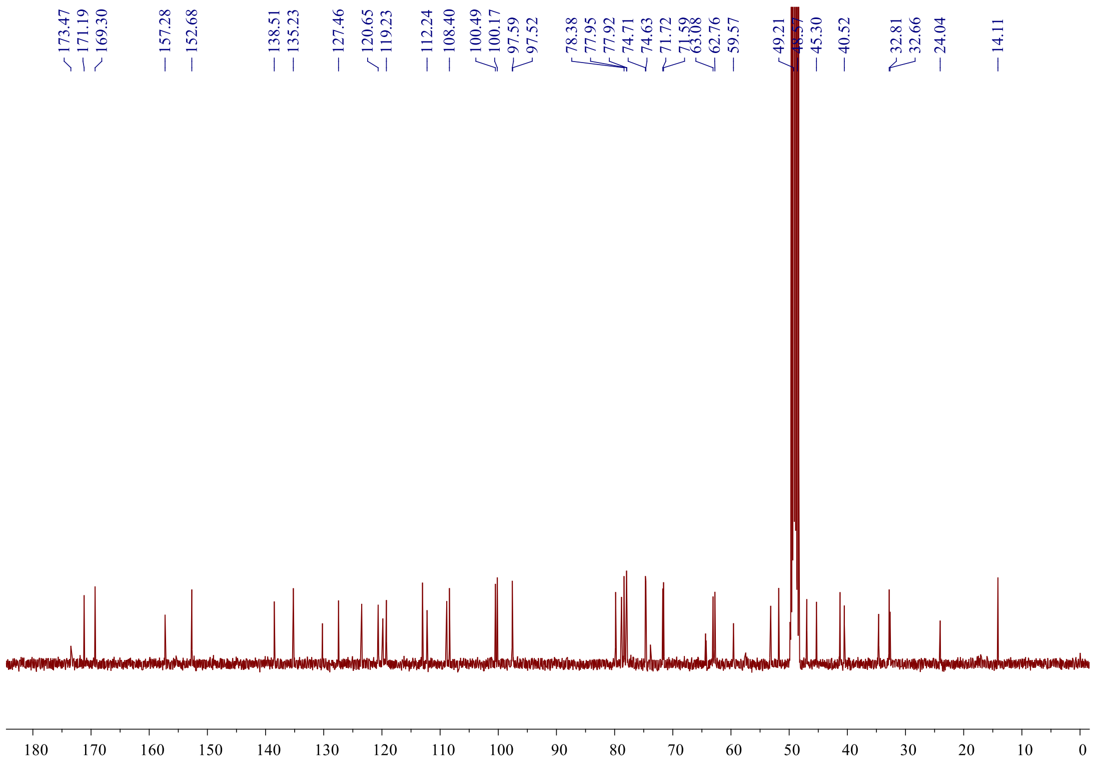

Figure S21. HSQC spectrum of compound **3** in CD_3OD (400 MHz).

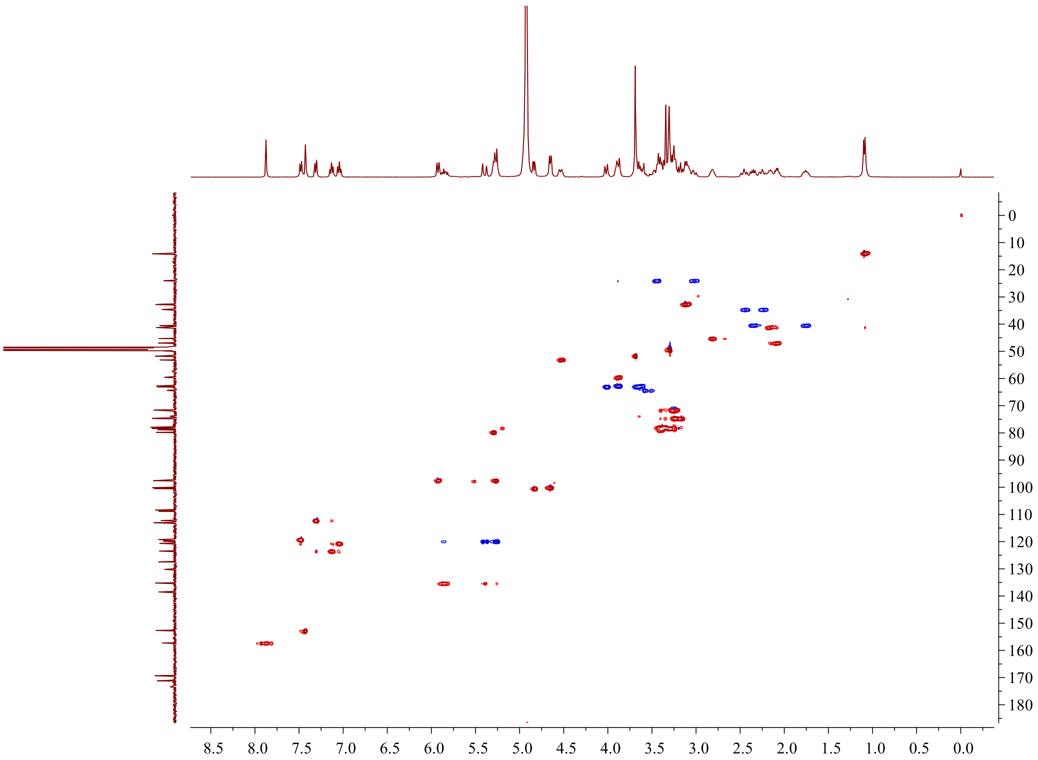

Figure S22. ^1H-^1H COSY spectrum of compound **3** in CD_3OD (400 MHz).

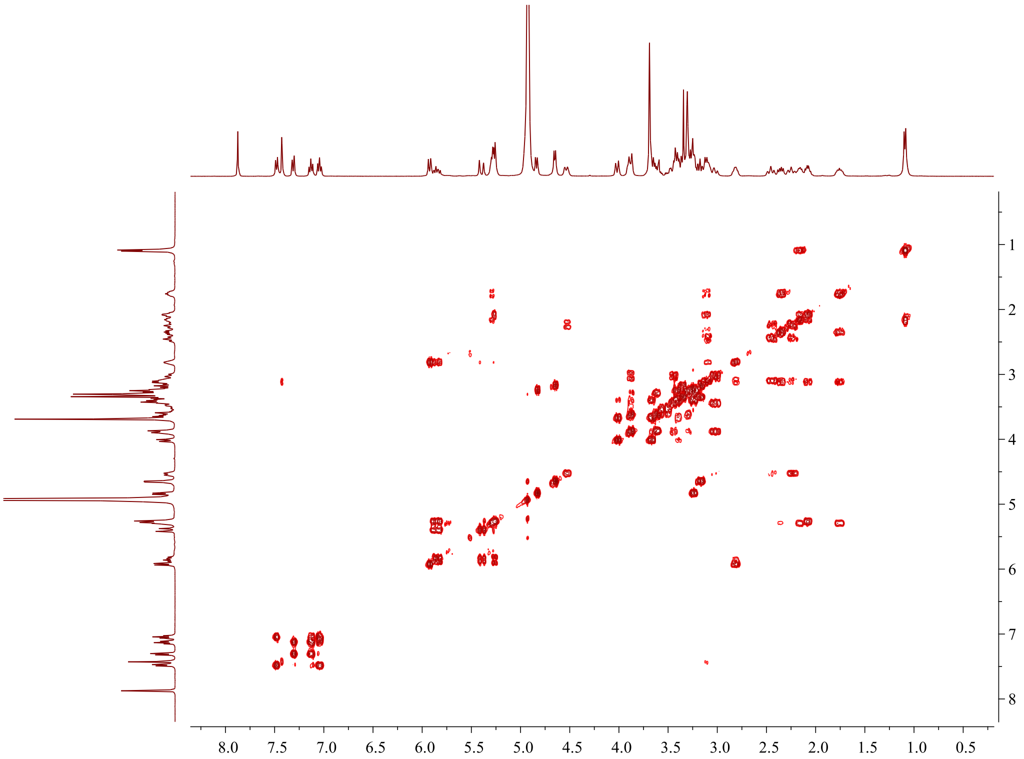

Figure S23. HMBC spectrum of compound **3** in CD_3OD (400 MHz).

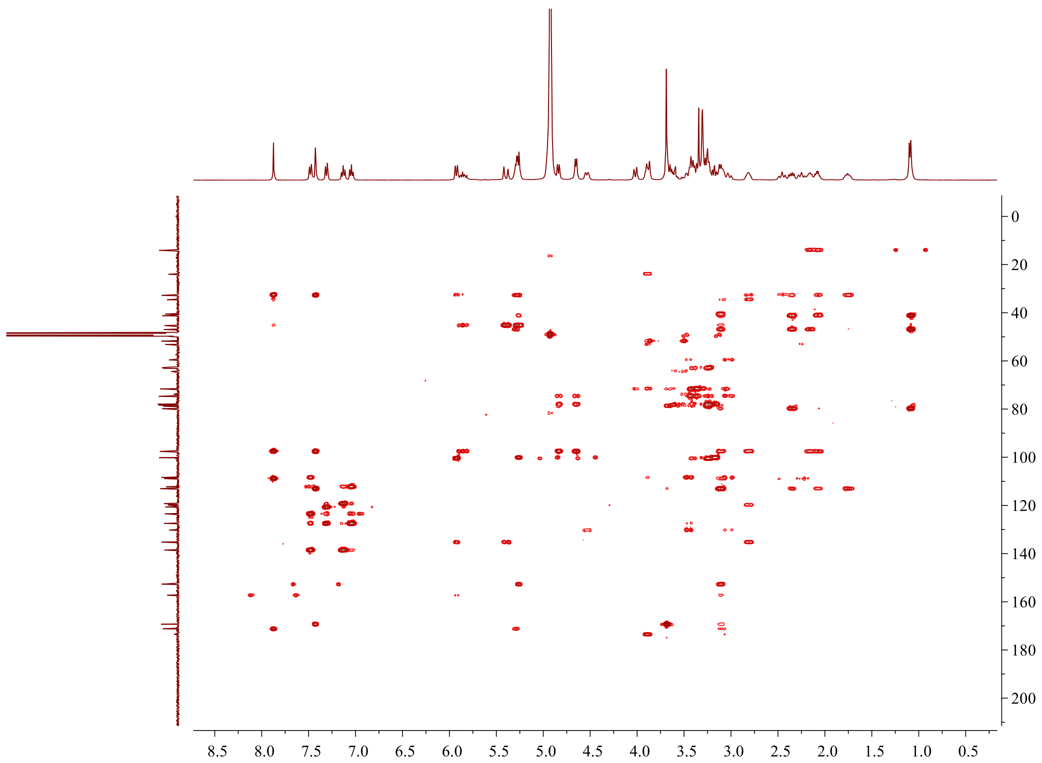

Figure S24. NOESY spectrum of compound **3** in CD_3OD (400 MHz).

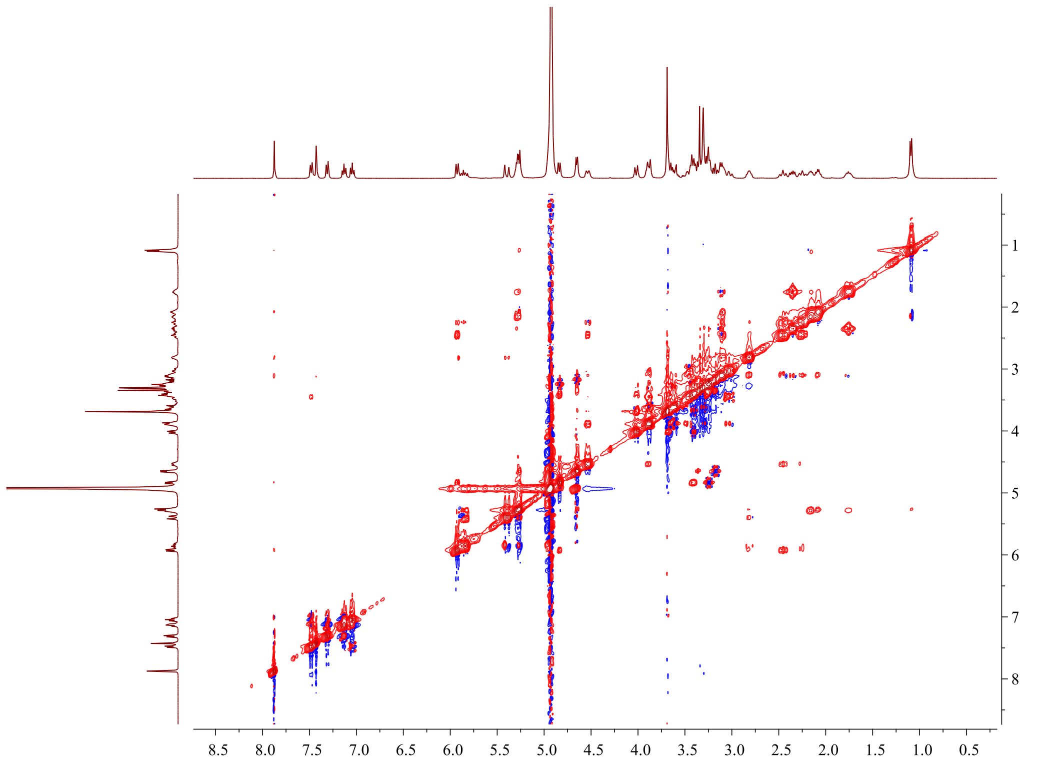

Figure S25. HRESIMS report of compound **3**.

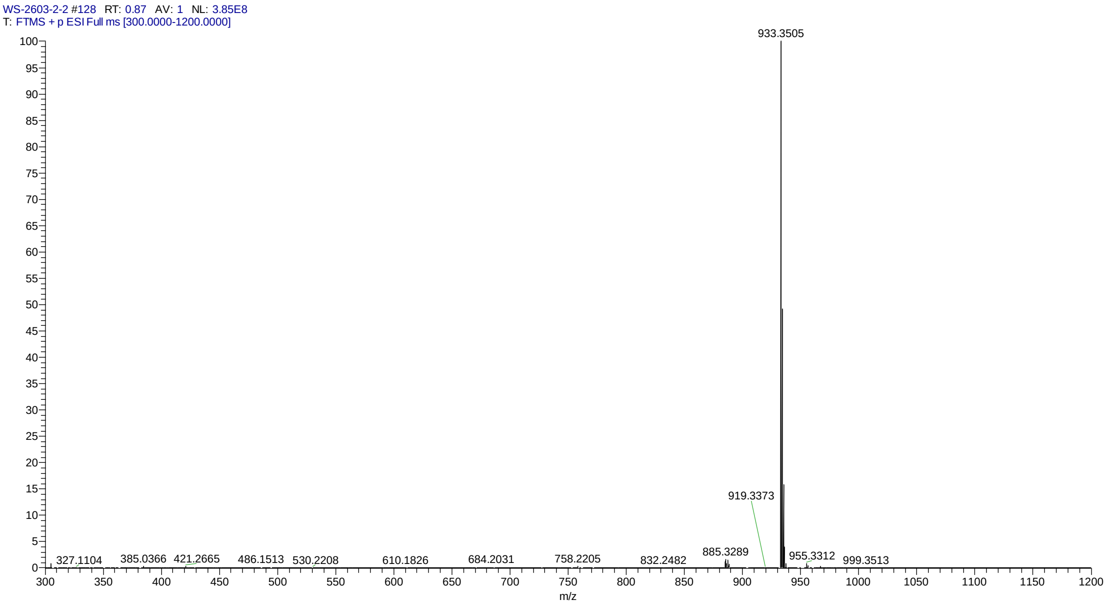

Figure S26. Experimental CD spectrum of **3** in MeOH.

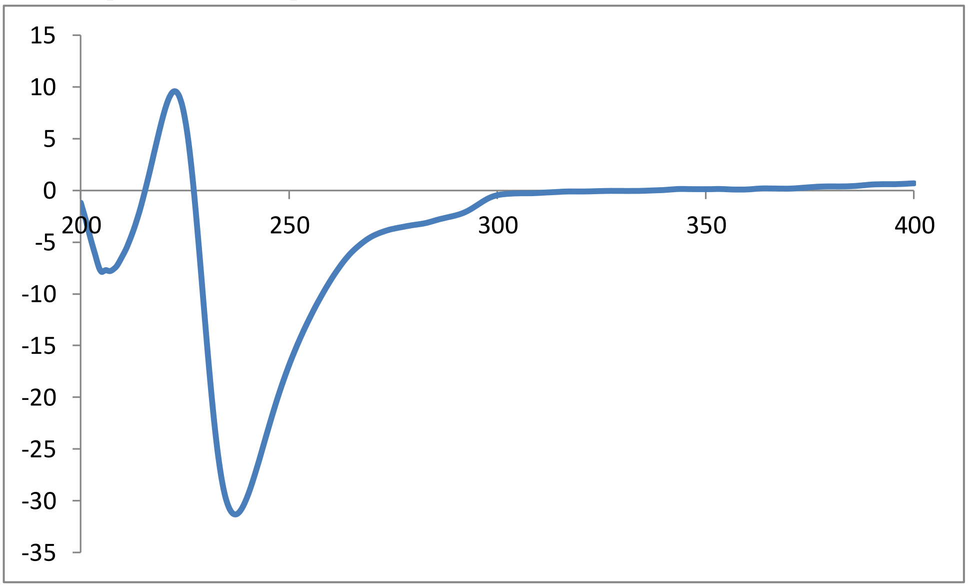

Figure S27. ^{1}H NMR spectrum of compound **4** in CD_3OD (400 MHz).

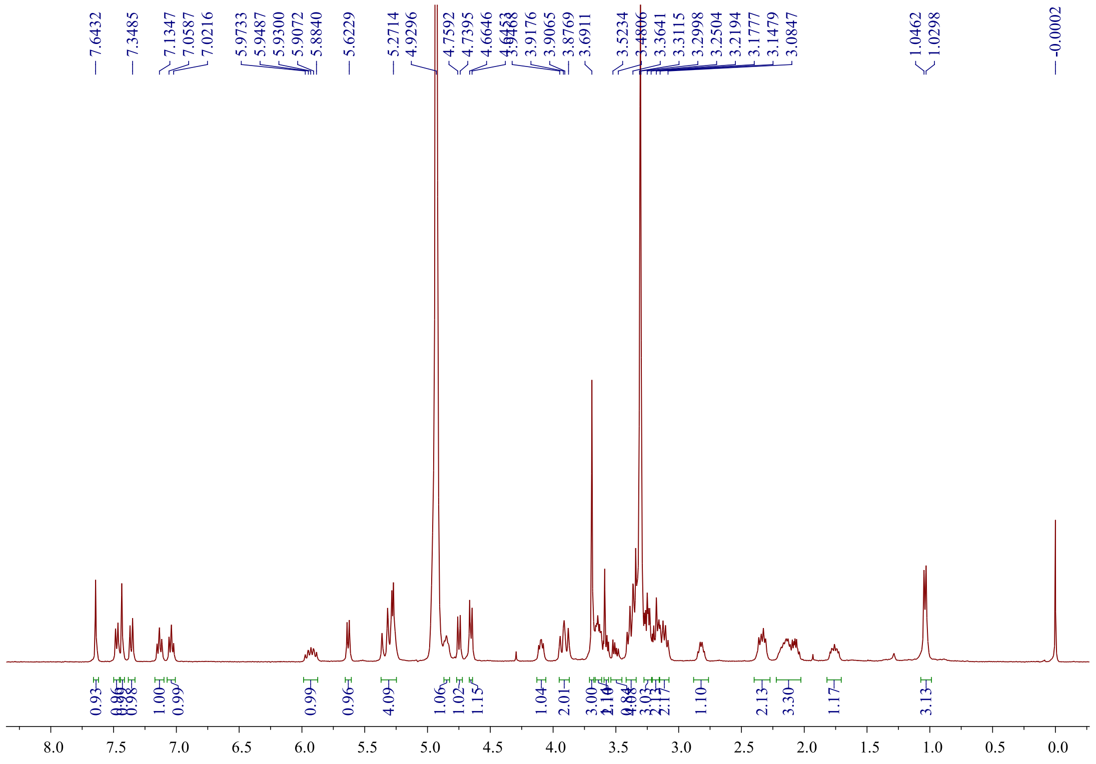

Figure S28. ^{13}C NMR spectrum of compound **4** in CD_3OD (100 MHz).

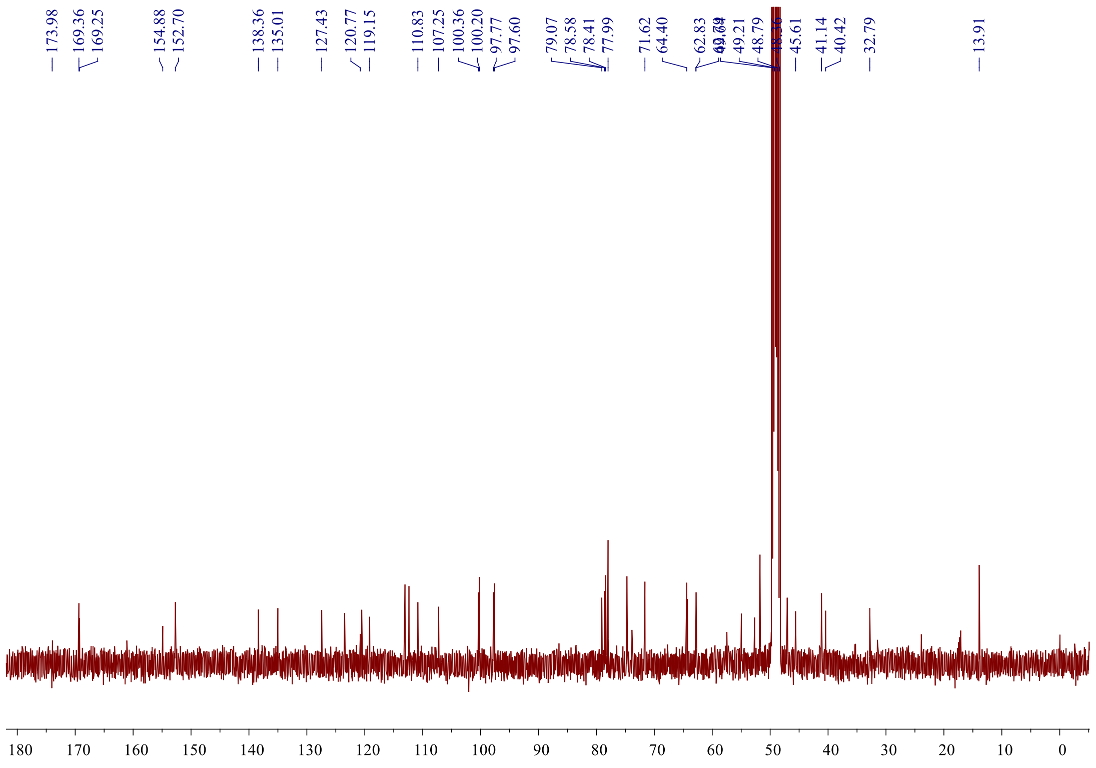

Figure S29. HSQC spectrum of compound **4** in CD_3OD (400 MHz).

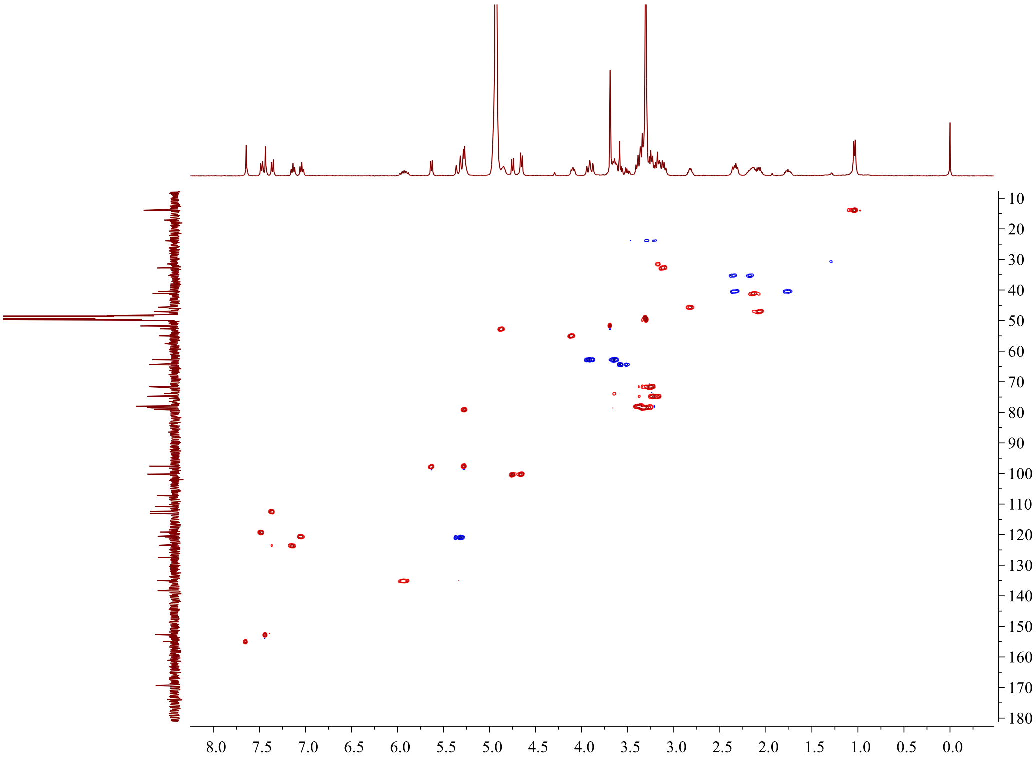

Figure S30. ^1H-^1H COSY spectrum of compound **4** in CD_3OD (400 MHz).

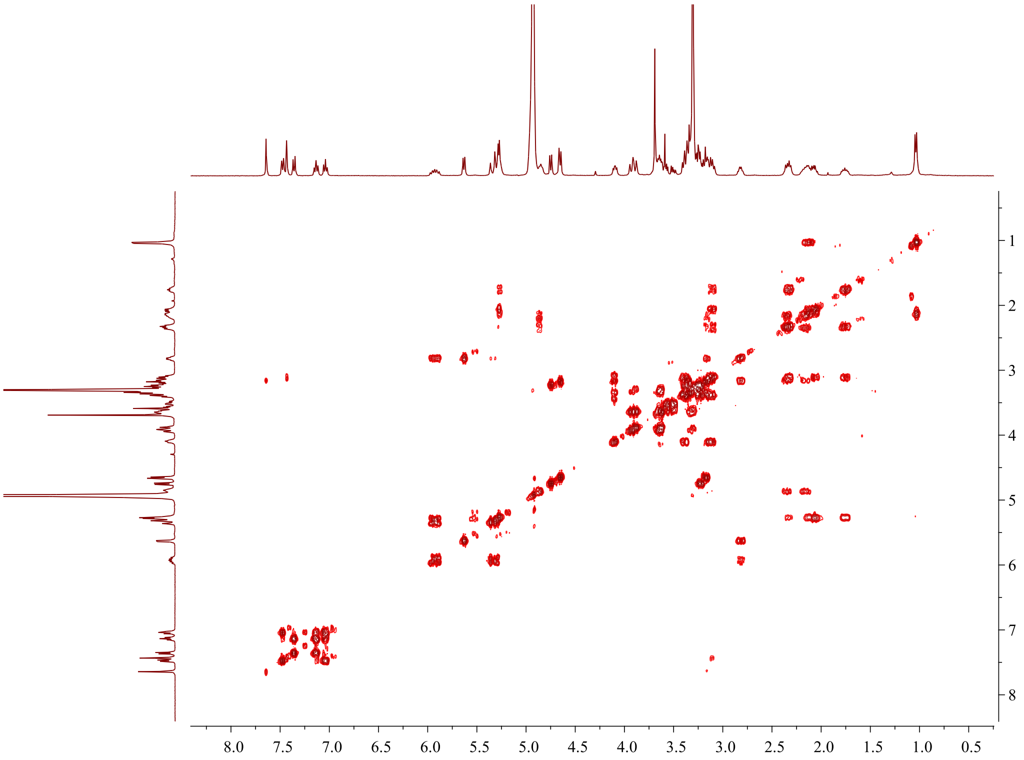

Figure S31. HMBC spectrum of compound **4** in CD_3OD (400 MHz).

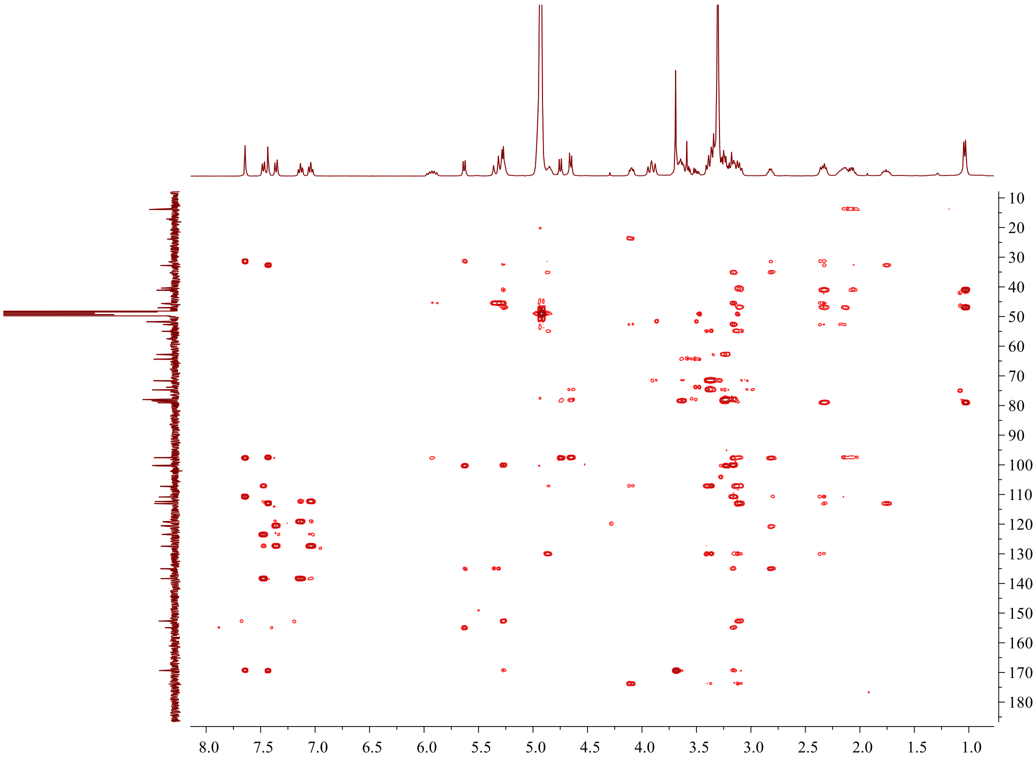

Figure S32. NOESY spectrum of compound **4** in CD_3OD (400 MHz).

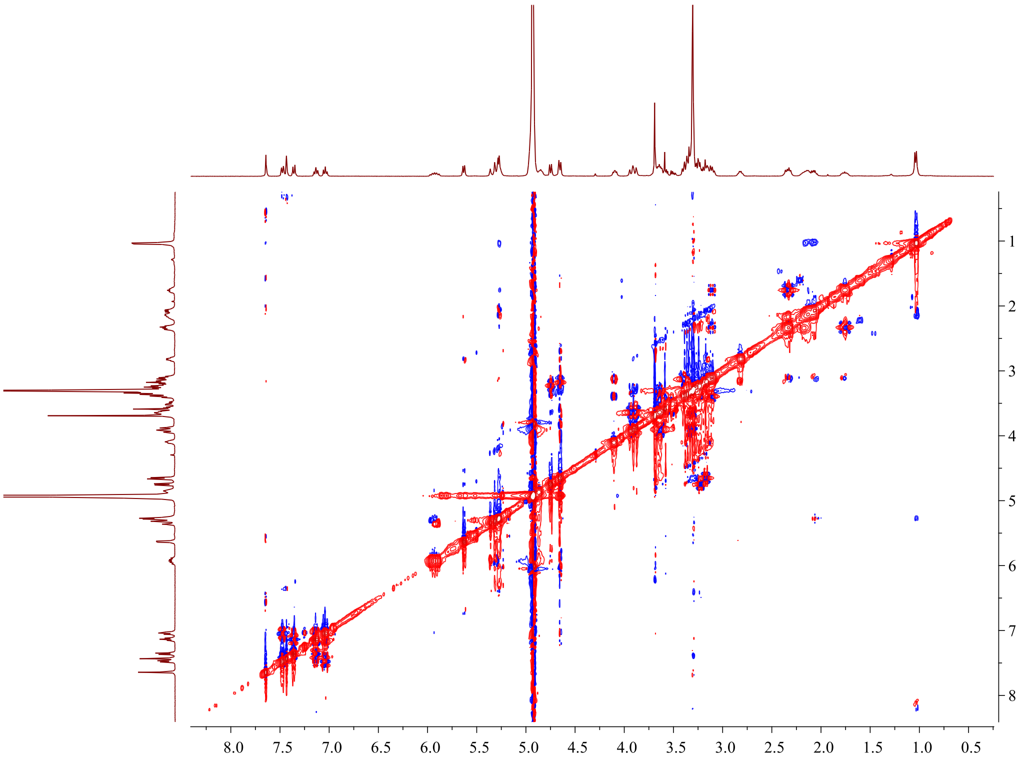

Figure S33. HRESIMS report of compound **4**.

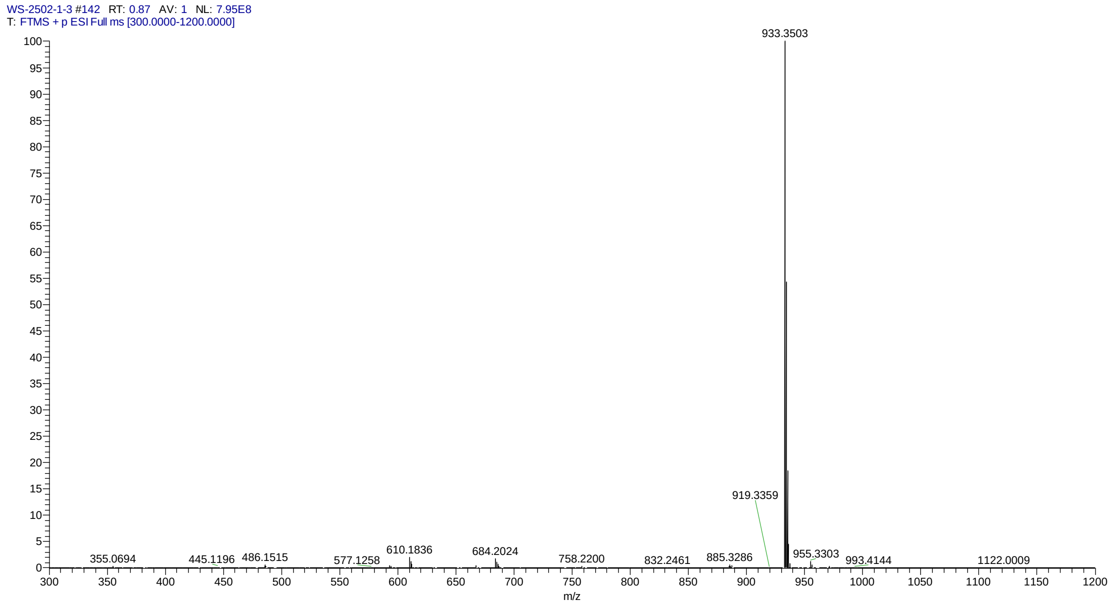

Figure S34. Experimental CD spectrum of **4** in MeOH.

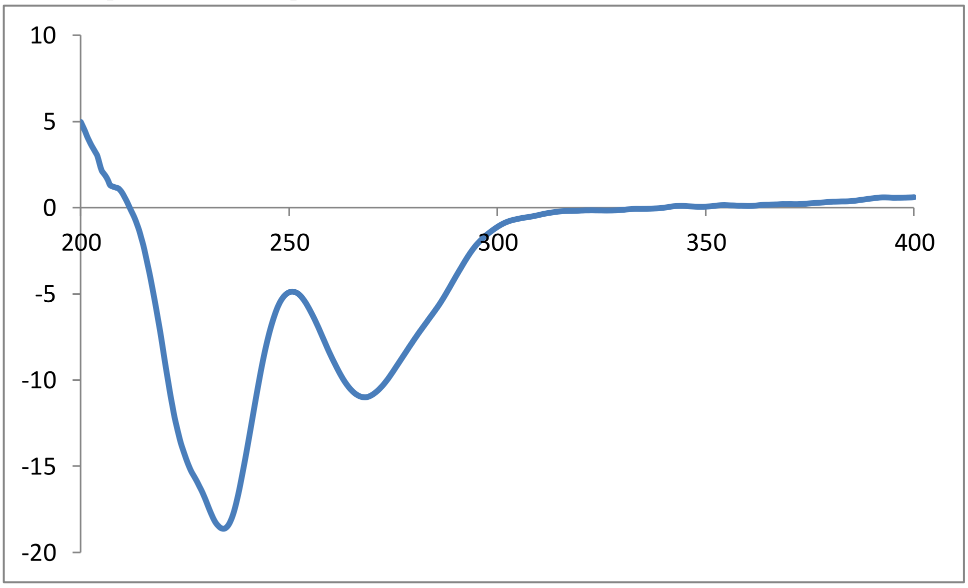

Figure S35. ^{1}H NMR spectrum of compound **5** in CD_3OD (400 MHz).

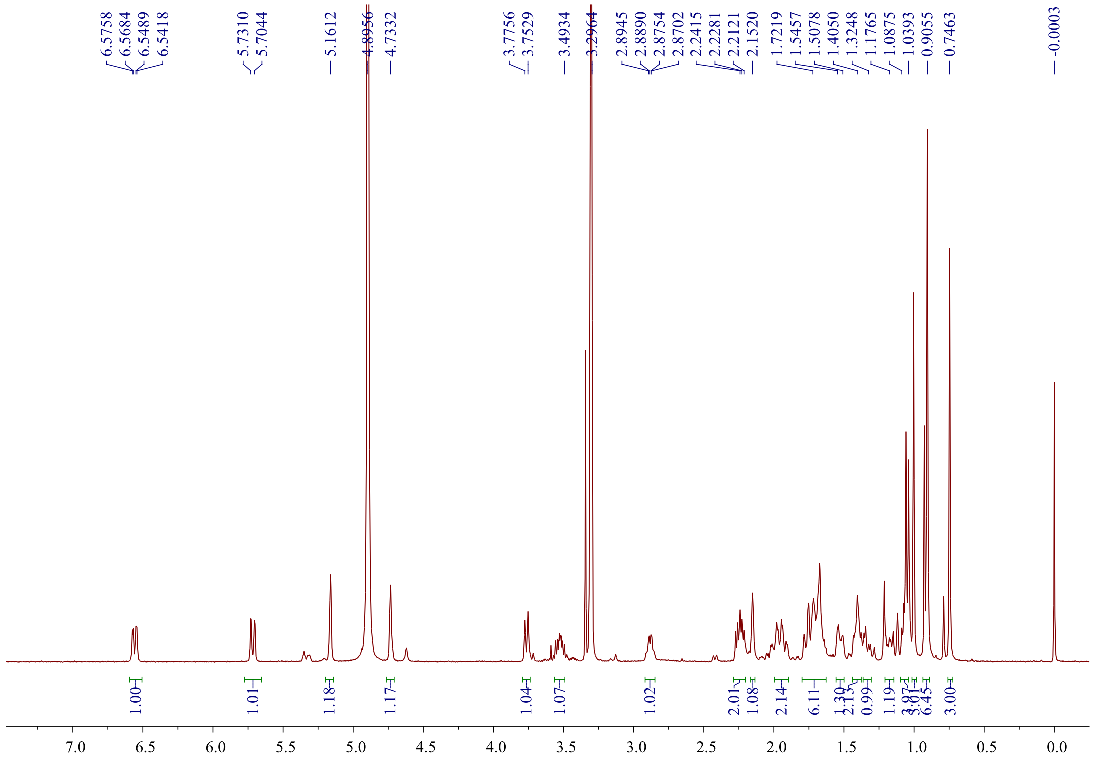

Figure S36. ^{13}C NMR spectrum of compound **5** in CD_3OD (100 MHz)

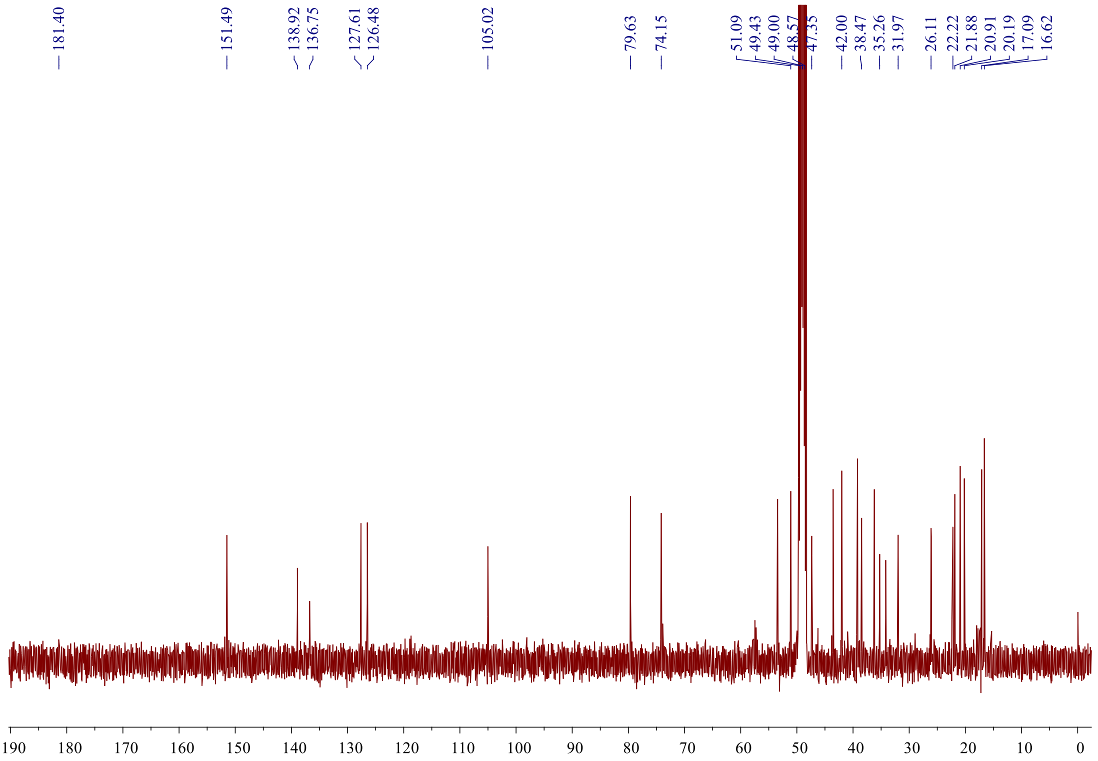

Figure S37. ^{1}H-^{1}H COSY spectrum of compound **5** in CD_3OD (400 MHz).

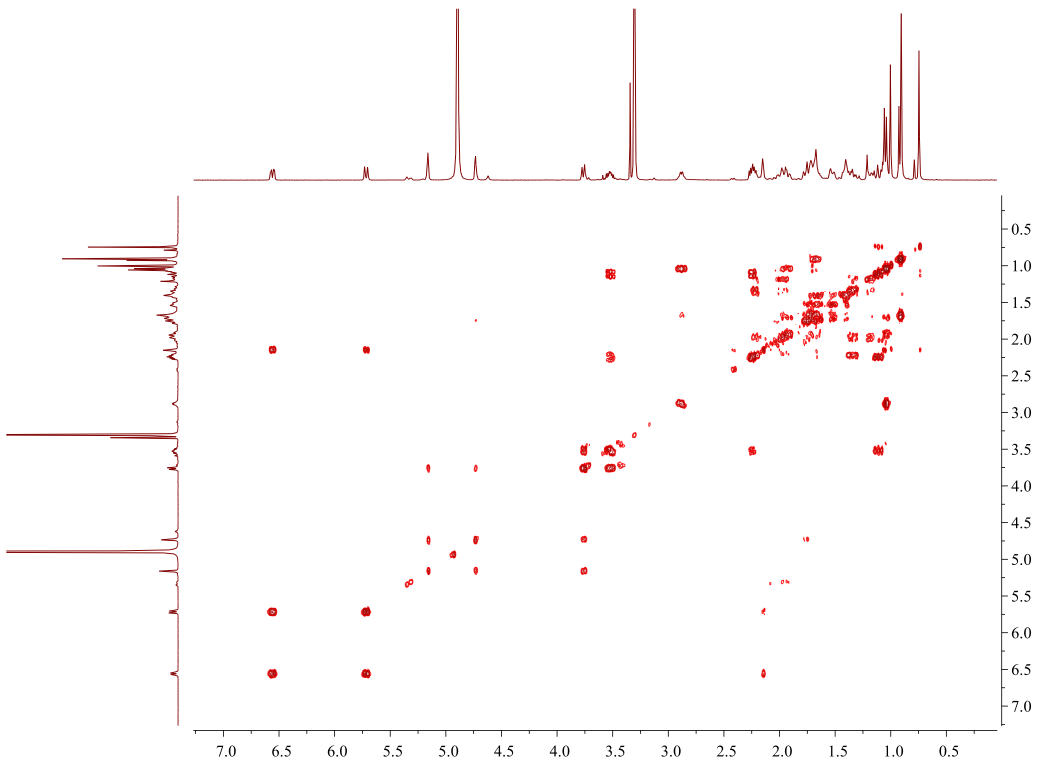

Figure S38. HMBC spectrum of compound **5** in CD_3OD (400 MHz).

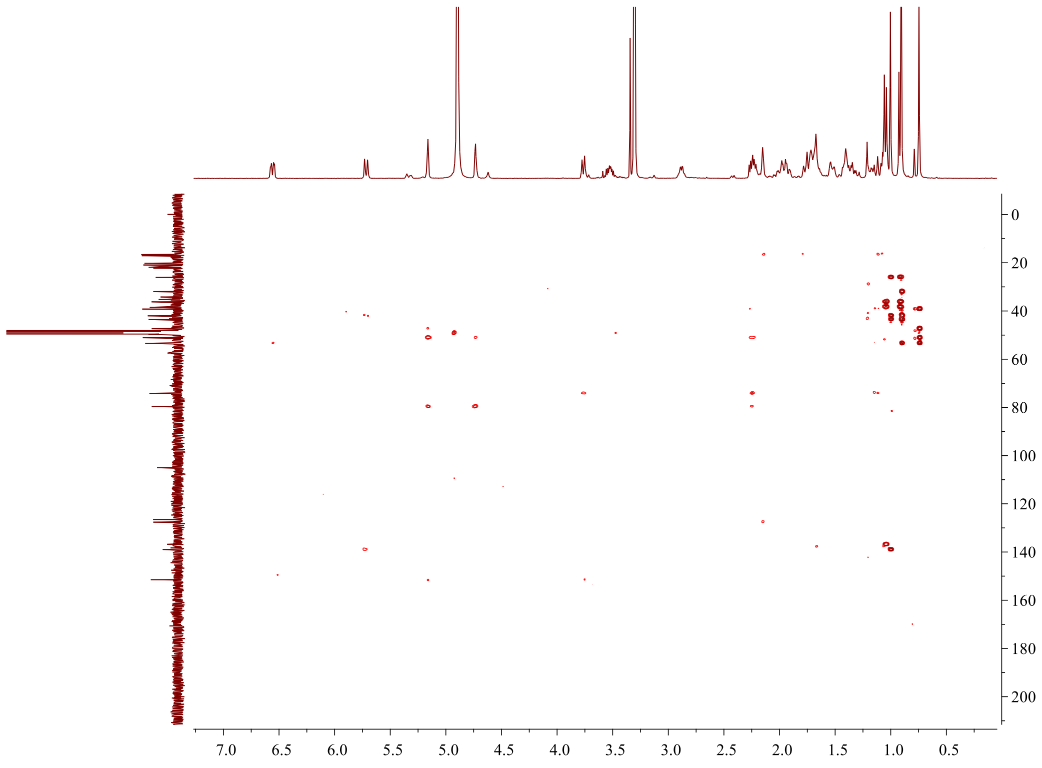

Figure S39. NOESY spectrum of compound **5** in CD_3OD (400 MHz).

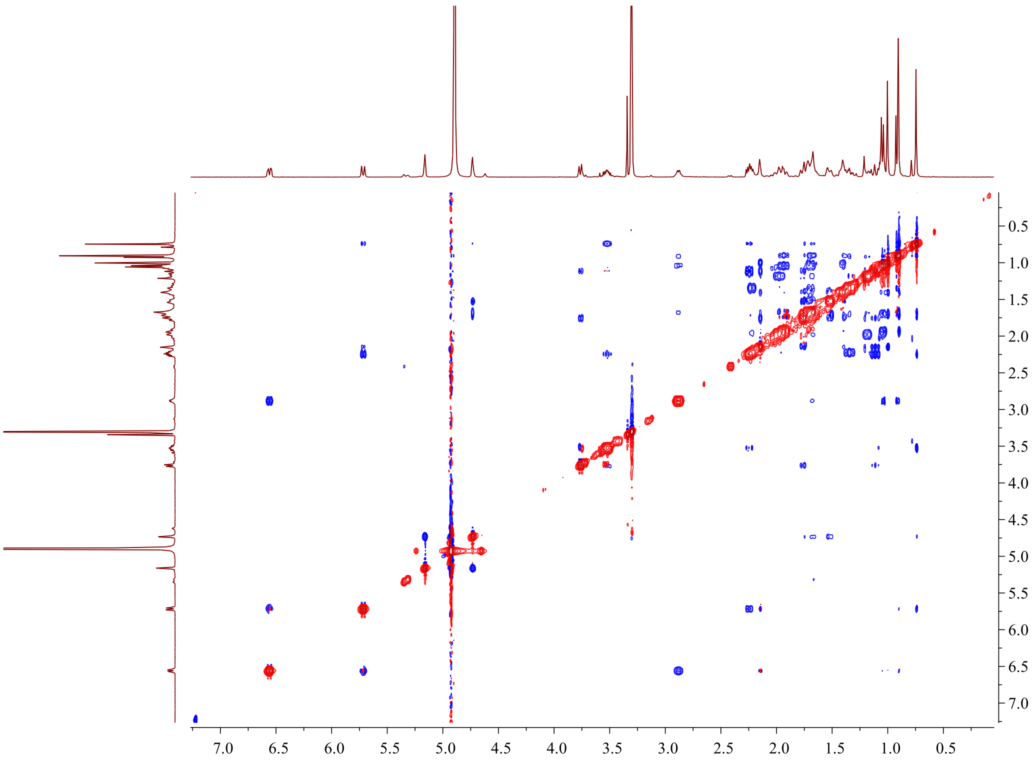

Figure S40. HRESIMS report of compound **5**.

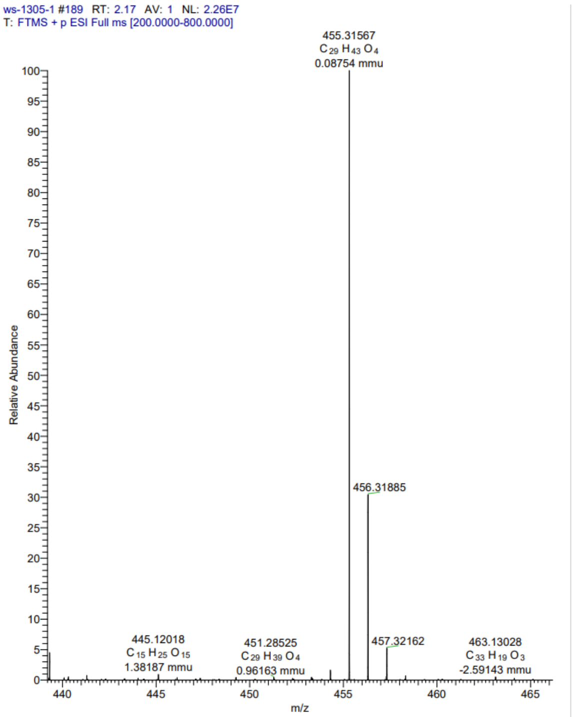

Figure S41. Experimental CD spectrum of **5** in MeOH.

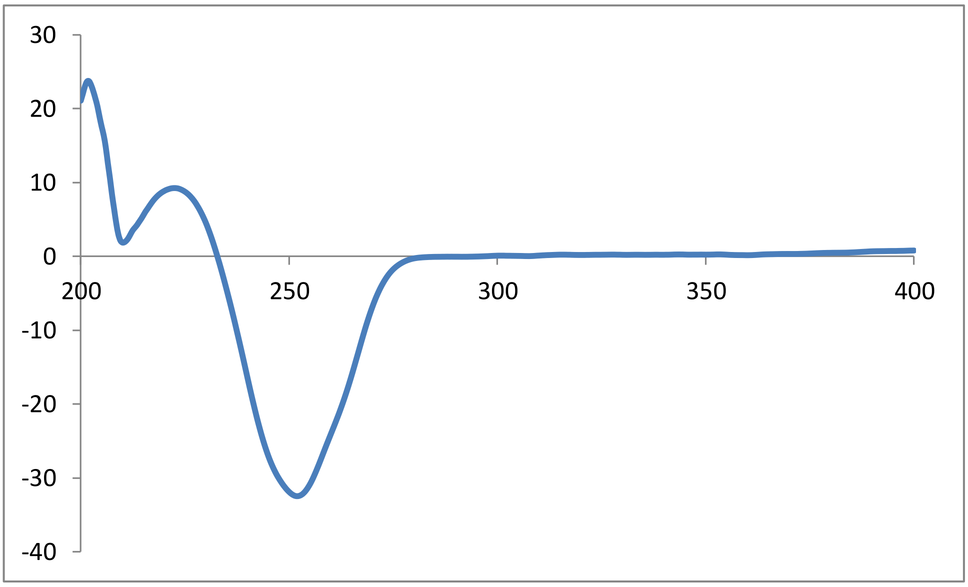

Figure S42. ^{1}H NMR spectrum of compound **6** in CD_3OD (400 MHz).

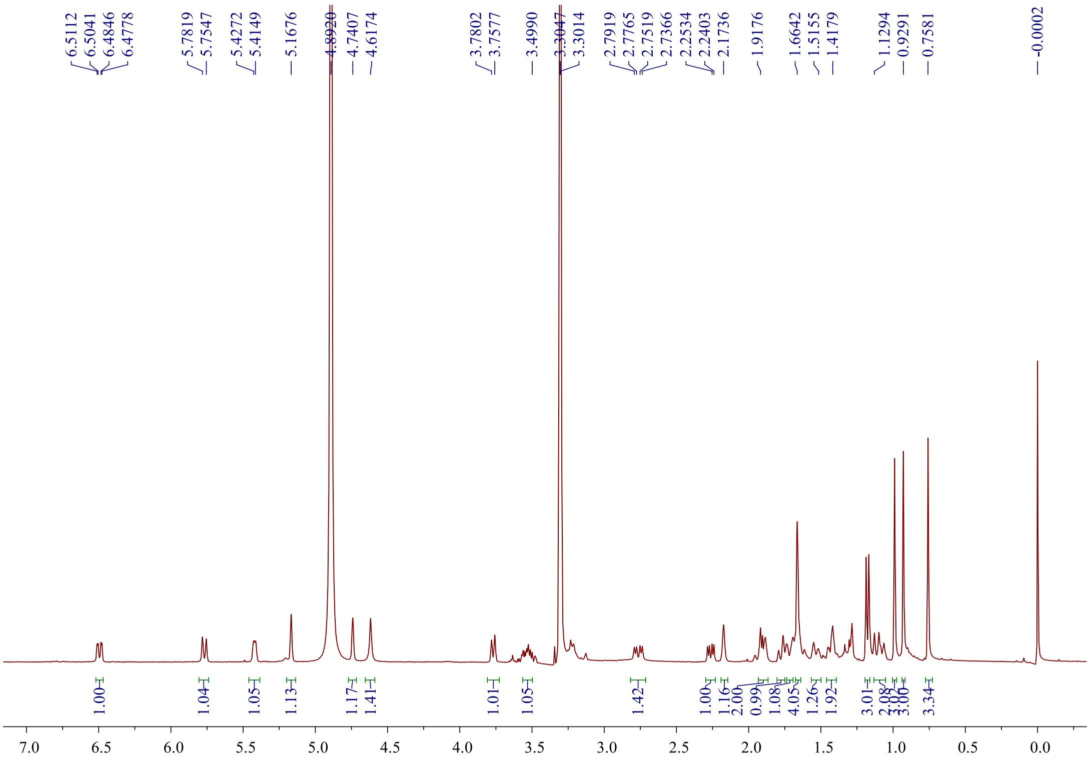

Figure S43. ^{13}C NMR spectrum of compound **6** in CD_3OD (100 MHz).

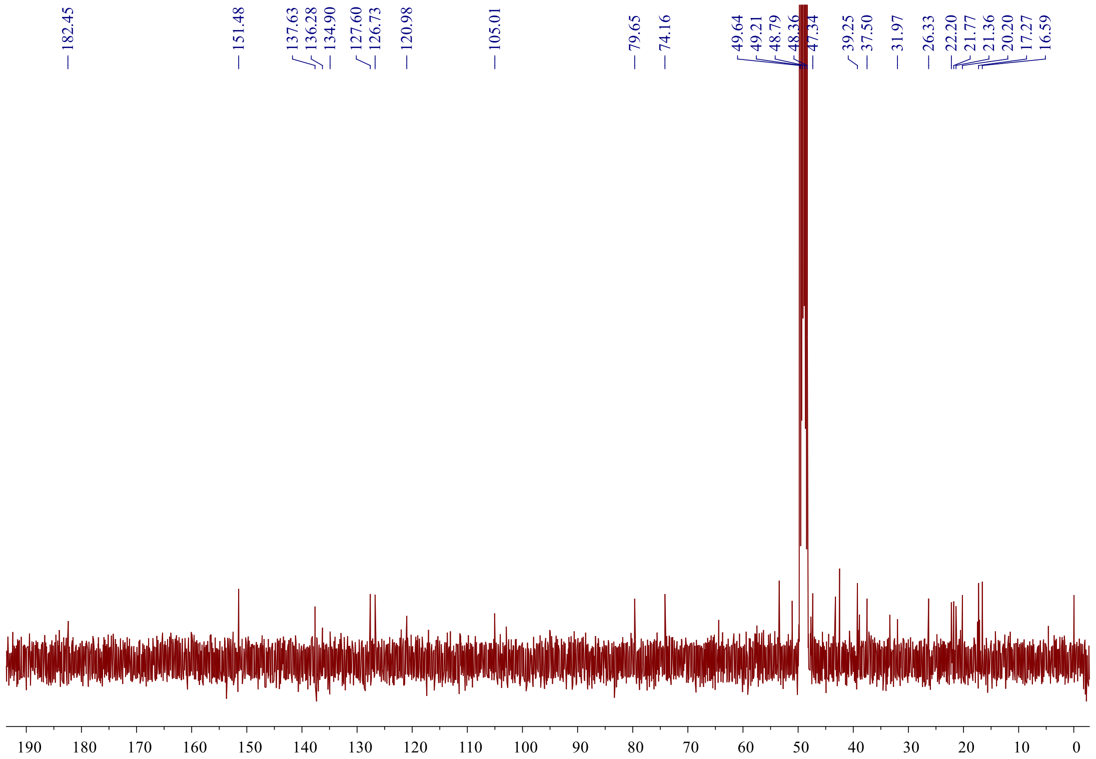

Figure S44. ^{1}H-^{1}H COSY spectrum of compound **6** in CD_3OD (400 MHz).

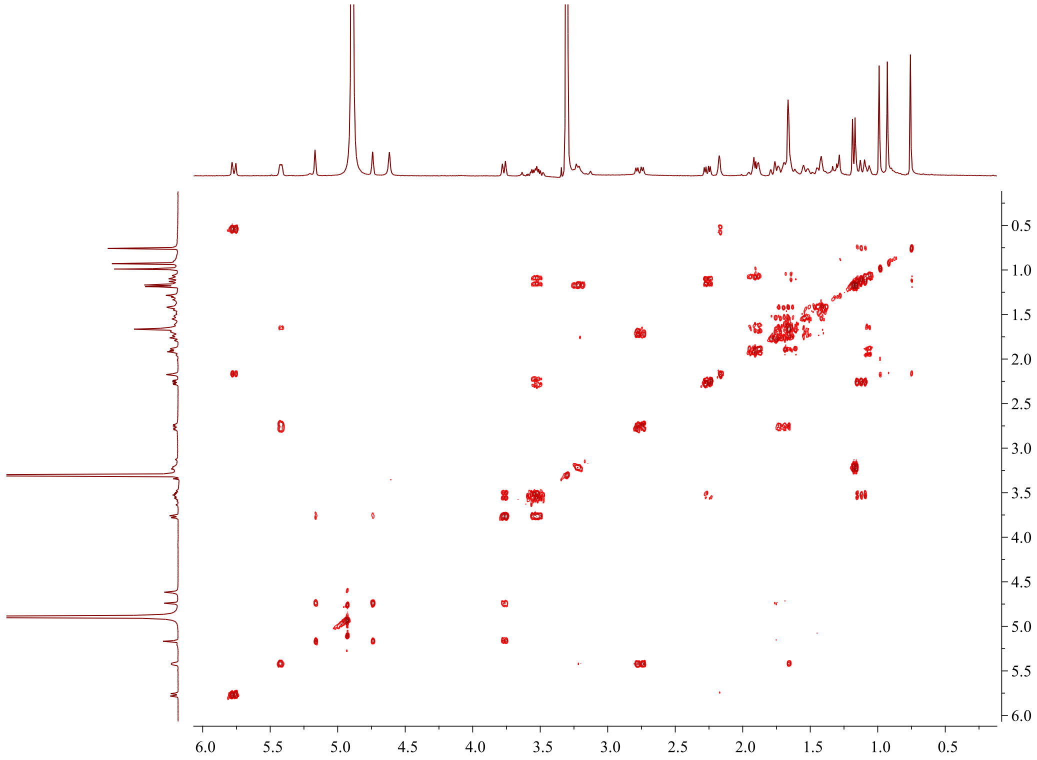

Figure S45. HMBC spectrum of compound **6** in CD_3OD (400 MHz).

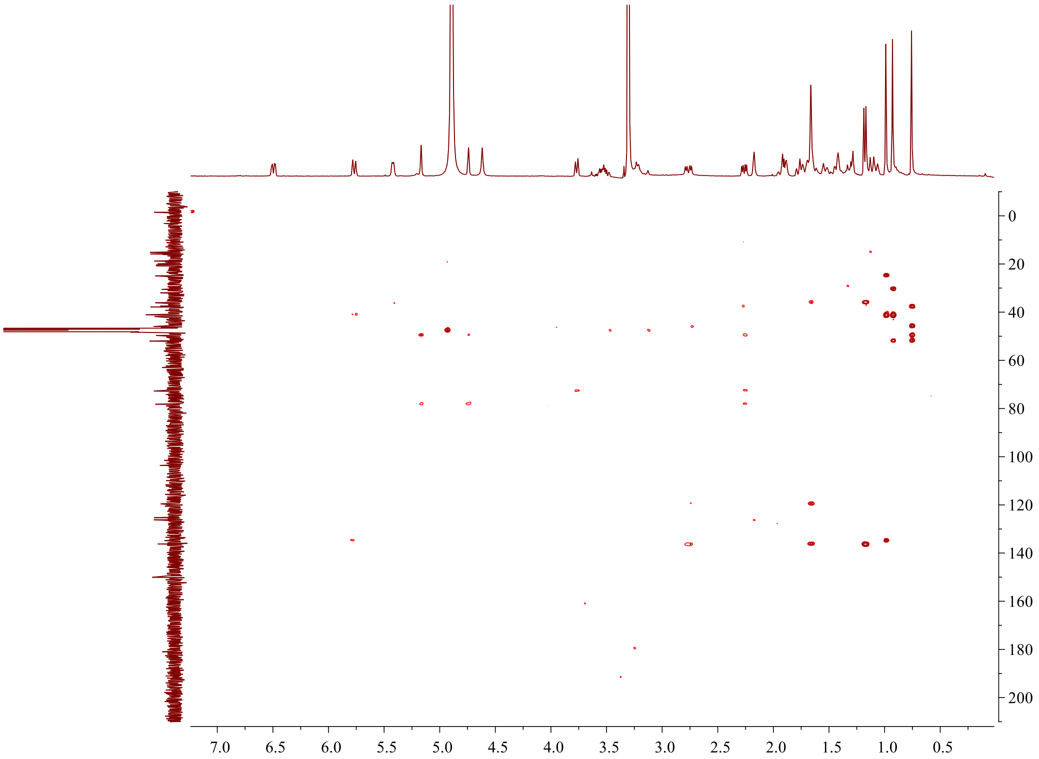

Figure S46. NOESY spectrum of compound **6** in CD_3OD (400 MHz).

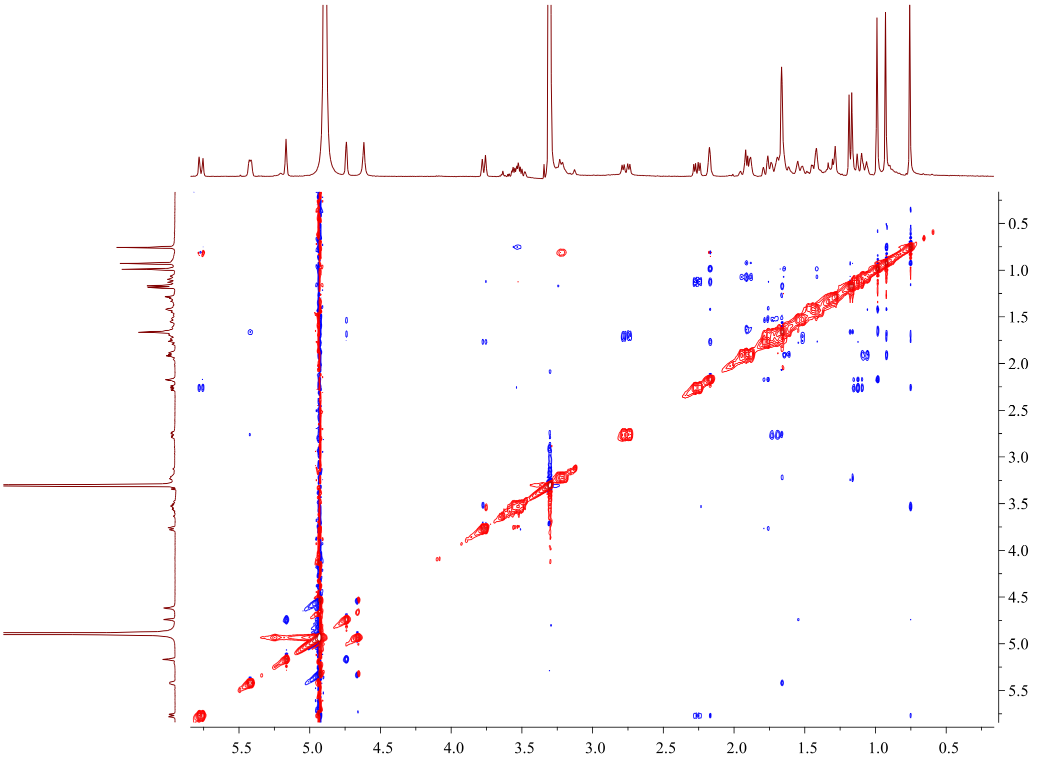

Figure S47. HRESIMS report of compound **6**.

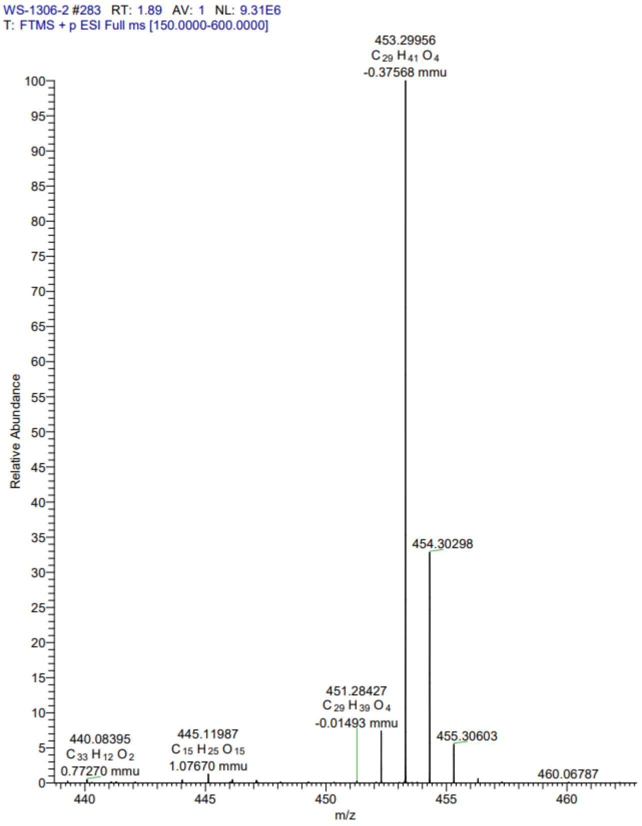

Supplement: Supplementary file 1 [file molecules-29-05980-s001.zip › molecules-3351587-supplementary.pdf]
